# Component retention in principal component analysis with application to cDNA microarray data

**DOI:** 10.1186/1745-6150-2-2

**Published:** 2007-01-17

**Authors:** Richard Cangelosi, Alain Goriely

**Affiliations:** 1 Department of Mathematics, University of Arizona, Tucson AZ85721, USA; 2 Program in Applied Mathematics, University of Arizona, Tucson AZ85721, USA; 3 BIO5 Institute, University of Arizona, Tucson AZ85721, USA

## Abstract

Shannon entropy is used to provide an estimate of the number of interpretable components in a principal component analysis. In addition, several ad hoc stopping rules for dimension determination are reviewed and a modification of the broken stick model is presented. The modification incorporates a test for the presence of an "effective degeneracy" among the subspaces spanned by the eigenvectors of the correlation matrix of the data set then allocates the total variance among subspaces. A summary of the performance of the methods applied to both published microarray data sets and to simulated data is given.

*This article was reviewed by Orly Alter, John Spouge (nominated by Eugene Koonin), David Horn and Roy Varshavsky (both nominated by O. Alter)*.

## 1 Background

Principal component analysis (PCA) is a 100 year old mathematical technique credited to Karl Pearson [1] and its properties as well as the interpretation of components have been investigated extensively [2-9]. The technique has found application in many diverse fields such as ecology, economics, psychology, meteorology, oceanography, and zoology. More recently it has been applied to the analysis of data obtained from cDNA microarray experiments [10-18]. cDNA microarray experiments provide a snapshot in time of gene expression levels across potentially thousands of genes and several time steps [19]. To assist in the data analysis, PCA (among other techniques) is generally employed as both a descriptive and data reduction technique. The focus of this letter will be on the latter.

In his development of PCA, Pearson [1] was interested in constructing a line or a plane that "best fits" a system of points in *q*-dimensional space. Geometrically, this amounts to repositioning the origin at the centroid of the points in *q*-dimensional space and then rotating the coordinate axes in such a way as to satisfy the maximal variance property. Statistically speaking, PCA represents a transformation of a set of *q *correlated variables into linear combinations of a set of *q *pair-wise uncorrelated variables called principal components. Components are constructed so that the first component explains the largest amount of total variance in the data and each subsequent component is constructed so as to explain the largest amount of the remaining variance while remaining uncorrelated with (orthogonal to) previously constructed components.

We define the dimension of the data set to be equal to the number of principal components. The set of *q *principal components is often reduced to a set of size *k*, where 1 ≤ *k *≪ *q*. The objective of dimension reduction is to make analysis and interpretation easier, while at the same time retaining most of the information (variation) contained in the data. Clearly, the closer the value of *k *is to *q *the better the PCA model will fit the data since more information has been retained, while the closer *k *is to 1, the simpler the model.

Many methods, both heuristic and statistically based, have been proposed to determine the number *k*, that is, the number of "meaningful" components. Some methods can be easily computed while others are computationally intensive. Methods include (among others): the broken stick model, the Kaiser-Guttman test, Log-Eigenvalue (LEV) diagram, Velicer's Partial Correlation Procedure, Cattell's SCREE test, cross-validation, bootstrapping techniques, cumulative percentage of total of variance, and Bartlett's test for equality of eigenvalues. For a description of these and other methods see [[7], Section 2.8] and [[9], Section 6.1]. For convenience, a brief overview of the techniques considered in this paper is given in the appendices.

Most techniques either suffer from an inherent subjectivity or have a tendency to under estimate or over estimate the true dimension of the data [20]. Ferré [21] concludes that there is no ideal solution to the problem of dimensionality in a PCA, while Jolliffe [9] notes "... it remains true that attempts to construct rules having more sound statistical foundations seem, at present, to offer little advantage over simpler rules in most circumstances." A comparison of the accuracy of certain methods based on real and simulated data can be found in [20-24].

Data reduction is frequently instrumental in revealing mathematical structure. The challenge is to balance the accuracy (or fit) of the model with ease of analysis and the potential loss of information. To confound matters, even random data may appear to have structure due to sampling variation. Karr and Martin [25] note that the percent variance attributed to principal components derived from real data may not be substantially greater than that derived from randomly generated data. They caution that most biologists could, given a set of random data, generate plausible "post-facto" explanations for high loadings in "variables." Basilevsky [26] cautions that it is not necessarily true that mathematical structure implies a physical process; however, the articles mentioned above provide examples of the successful implementation of the technique.

In this report, we apply nine ad-hoc methods to previously published and publicly available microarray data and summarize the results. We also introduce a modification of the broken stick model which incorporates the notion of degenerate subspaces in component retention. Finally, we introduce and include in the summary a novel application of statistical entropy to provide a new heuristic measure of the number of interpretable components.

## 2 Mathematical methods

### 2.1 Principal component analysis

Each principal component represents a linear combination of the original variables with the first principal component defined as the linear combination with maximal sample variance among all linear combinations of the variables. The next principal component represents the linear combination that explains the maximal sample variance that remains unexplained by the first with the additional condition that it is orthogonal to the first [27]. Each subsequent component is determined in a similar fashion. If we have a *q*-dimensional space, we expect to have *q *principal components due to sampling variation.

The following derivation can be found in [[27], pp. 373–374], Jolliffe [9] or Basilevsky [26]. Let *X *be a (*p *× *q*) matrix that contains the observed expression of the *i*-th gene in its *i*-th row. Denote by *g*_*i *_the *i*-th observation and let *S *be the sample covariance matrix of *X*. For a particular observation *g*_*i*_, we seek

*z *= *a*_1_*g*_*i*,1 _+ *a*_2_*g*_*i*,2 _+ ... + *a*_*p*_*g*_*i,p *_= a→Tg→i
 MathType@MTEF@5@5@+=feaafiart1ev1aaatCvAUfKttLearuWrP9MDH5MBPbIqV92AaeXatLxBI9gBaebbnrfifHhDYfgasaacH8akY=wiFfYdH8Gipec8Eeeu0xXdbba9frFj0=OqFfea0dXdd9vqai=hGuQ8kuc9pgc9s8qqaq=dirpe0xb9q8qiLsFr0=vr0=vr0dc8meaabaqaciaacaGaaeqabaqabeGadaaakeaacuWGHbqygaWcamaaCaaaleqabaGaemivaqfaaOGafm4zaCMbaSaadaWgaaWcbaGaemyAaKgabeaaaaa@3261@     (1)

such that var(*z*) = var(a→Tg→i
 MathType@MTEF@5@5@+=feaafiart1ev1aaatCvAUfKttLearuWrP9MDH5MBPbIqV92AaeXatLxBI9gBaebbnrfifHhDYfgasaacH8akY=wiFfYdH8Gipec8Eeeu0xXdbba9frFj0=OqFfea0dXdd9vqai=hGuQ8kuc9pgc9s8qqaq=dirpe0xb9q8qiLsFr0=vr0=vr0dc8meaabaqaciaacaGaaeqabaqabeGadaaakeaacuWGHbqygaWcamaaCaaaleqabaGaemivaqfaaOGafm4zaCMbaSaadaWgaaWcbaGaemyAaKgabeaaaaa@3261@) is maximal subject to a→Ta→
 MathType@MTEF@5@5@+=feaafiart1ev1aaatCvAUfKttLearuWrP9MDH5MBPbIqV92AaeXatLxBI9gBaebbnrfifHhDYfgasaacH8akY=wiFfYdH8Gipec8Eeeu0xXdbba9frFj0=OqFfea0dXdd9vqai=hGuQ8kuc9pgc9s8qqaq=dirpe0xb9q8qiLsFr0=vr0=vr0dc8meaabaqaciaacaGaaeqabaqabeGadaaakeaacuWGHbqygaWcamaaCaaaleqabaGaemivaqfaaOGafmyyaeMbaSaaaaa@30CE@ = 1. That is, we maximize the expression

a→TSa→−λ(a→Ta→−1)     (2)
 MathType@MTEF@5@5@+=feaafiart1ev1aaatCvAUfKttLearuWrP9MDH5MBPbIqV92AaeXatLxBI9gBaebbnrfifHhDYfgasaacH8akY=wiFfYdH8Gipec8Eeeu0xXdbba9frFj0=OqFfea0dXdd9vqai=hGuQ8kuc9pgc9s8qqaq=dirpe0xb9q8qiLsFr0=vr0=vr0dc8meaabaqaciaacaGaaeqabaqabeGadaaakeaacuWGHbqygaWcamaaCaaaleqabaGaemivaqfaaOGaem4uamLafmyyaeMbaSaacqGHsisliiGacqWF7oaBdaqadaqaaiqbdggaHzaalaWaaWbaaSqabeaacqWGubavaaGccuWGHbqygaWcaiabgkHiTiabigdaXaGaayjkaiaawMcaaiaaxMaacaWLjaWaaeWaaeaacqaIYaGmaiaawIcacaGLPaaaaaa@3FEC@

where *λ *is a Lagrange multiplier. Differentiating with respect to a→
 MathType@MTEF@5@5@+=feaafiart1ev1aaatCvAUfKttLearuWrP9MDH5MBPbIqV92AaeXatLxBI9gBaebbnrfifHhDYfgasaacH8akY=wiFfYdH8Gipec8Eeeu0xXdbba9frFj0=OqFfea0dXdd9vqai=hGuQ8kuc9pgc9s8qqaq=dirpe0xb9q8qiLsFr0=vr0=vr0dc8meaabaqaciaacaGaaeqabaqabeGadaaakeaacuWGHbqygaWcaaaa@2E09@ leads to the familiar eigenvalue problem

Sa→−λa→=0     (3)
 MathType@MTEF@5@5@+=feaafiart1ev1aaatCvAUfKttLearuWrP9MDH5MBPbIqV92AaeXatLxBI9gBaebbnrfifHhDYfgasaacH8akY=wiFfYdH8Gipec8Eeeu0xXdbba9frFj0=OqFfea0dXdd9vqai=hGuQ8kuc9pgc9s8qqaq=dirpe0xb9q8qiLsFr0=vr0=vr0dc8meaabaqaciaacaGaaeqabaqabeGadaaakeaacqWGtbWucuWGHbqygaWcaiabgkHiTGGaciab=T7aSjqbdggaHzaalaGaeyypa0JaeGimaaJaaCzcaiaaxMaadaqadaqaaiabiodaZaGaayjkaiaawMcaaaaa@38F2@

So *λ *is an eigenvalue of *S *and a→TSa→=a→Tλa→=λa→Ta→=λ     (4)
 MathType@MTEF@5@5@+=feaafiart1ev1aaatCvAUfKttLearuWrP9MDH5MBPbIqV92AaeXatLxBI9gBaebbnrfifHhDYfgasaacH8akY=wiFfYdH8Gipec8Eeeu0xXdbba9frFj0=OqFfea0dXdd9vqai=hGuQ8kuc9pgc9s8qqaq=dirpe0xb9q8qiLsFr0=vr0=vr0dc8meaabaqaciaacaGaaeqabaqabeGadaaakeaacuWGHbqygaWcamaaCaaaleqabaGaemivaqfaaOGaem4uamLafmyyaeMbaSaacqGH9aqpcuWGHbqygaWcamaaCaaaleqabaGaemivaqfaaGGacOGae83UdWMafmyyaeMbaSaacqGH9aqpcqWF7oaBcuWGHbqygaWcamaaCaaaleqabaGaemivaqfaaOGafmyyaeMbaSaacqGH9aqpcqWF7oaBcaWLjaGaaCzcamaabmaabaGaeGinaqdacaGLOaGaayzkaaaaaa@462F@ is its corresponding eigenvector. Since

a→TSa→=a→Tλa→=λa→Ta→=λ     (4)
 MathType@MTEF@5@5@+=feaafiart1ev1aaatCvAUfKttLearuWrP9MDH5MBPbIqV92AaeXatLxBI9gBaebbnrfifHhDYfgasaacH8akY=wiFfYdH8Gipec8Eeeu0xXdbba9frFj0=OqFfea0dXdd9vqai=hGuQ8kuc9pgc9s8qqaq=dirpe0xb9q8qiLsFr0=vr0=vr0dc8meaabaqaciaacaGaaeqabaqabeGadaaakeaacuWGHbqygaWcamaaCaaaleqabaGaemivaqfaaOGaem4uamLafmyyaeMbaSaacqGH9aqpcuWGHbqygaWcamaaCaaaleqabaGaemivaqfaaGGacOGae83UdWMafmyyaeMbaSaacqGH9aqpcqWF7oaBcuWGHbqygaWcamaaCaaaleqabaGaemivaqfaaOGafmyyaeMbaSaacqGH9aqpcqWF7oaBcaWLjaGaaCzcamaabmaabaGaeGinaqdacaGLOaGaayzkaaaaaa@462F@

we see that to maximize the expression we should choose the largest eigenvalue and its associated eigenvector. Proceed in a similar fashion to determine all *q *eigenvalues and eigenvectors.

#### 2.1.1 Data preprocessing

Data obtained from cDNA microarray experiments are frequently "polished" or pre-processed. This may include, but is not limited to: log transformations, the use of weights and metrics, mean centering of rows (genes) or columns (arrays), and normalization, which sets the magnitude of a row or column vector equal to one. The term data preprocessing varies from author to author and its merits and implications can be found in [28-32].

**Figure 1:**
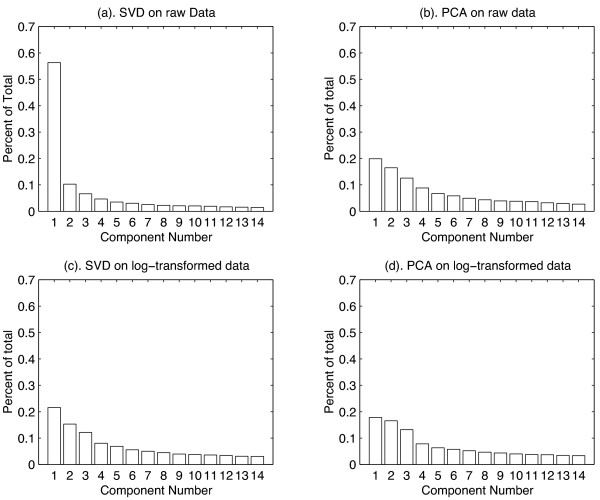
**SVD and PCA**. (a) SVD performed on the elutriation data set; (b) PCA on the elutriation data set; (c) SVD on the log base two transformed data; (d) PCA on the log base two data.

It is important to note that such operations will affect the eigensystem of the data matrix. A simple example is provided by comparing the singular spectrum from a singular value decomposition (SVD) with that of a traditional PCA. Note that PCA can be considered as a special case of singular value decomposition [32]. In SVD one computes the eigensystem of *X*^*T*^*X*, where the *p *× *q *matrix *X *contains the gene expression data. In PCA one computes the eigensystem of *S *= *M*^*T*^*M*/(*p *- 1), where *M *equals the re-scaled and column centered (column means are zero) matrix *X*. The matrix *S *is recognized as the sample covariance matrix of the data. Figure 1 illustrates the eigenvalues (expressed as a percent of total dispersion) obtained from a PCA and an SVD on both the raw and log base-two transformed [33] elutriation data set of the budding yeast *Saccharomyces cerevisiae *[34]. Note the robustness of PCA. In Figure 1.a, which is an SVD performed on the raw data, we see the dominance of the first mode. In general, the further the mean is from the origin, the larger the largest singular value will be in a SVD relative to the others [7].

### 2.2 Broken stick model

The so-called broken stick model has been referred to as a resource apportionment model [35] and was first presented as such by MacArthur [36] in the study of the structure of animal communities, specifically, bird species from various regions. Frontier [37] proposed comparing eigenvalues from a PCA to values given by the broken stick distribution. The apportioned resource is the total variance of the data set (the variance is considered a resource shared among the principal components). Since each eigenvalue of a PCA represents a measure of each components' variance, a component is retained if its associated eigenvalue is larger than the value given by the broken stick distribution. An example of broken stick distribution with a plot can be found in Section 3.2.

As with all methods currently in use, the broken stick model has drawbacks and advantages. Since the model does not consider sample size, Franklin *et al. *[23] contends that the broken stick distribution cannot really model sampling distributions of eigenvalues. The model also has a tendency to underestimate the dimension of the data [20-22]. However, Jackson [20] claims that the broken stick model accurately determined the correct dimensionality in three of the four patterned matrices used in his study, giving underestimates in the other. He reported that overall, the model was one of the two most accurate under consideration. Bartkowiak [22] claims that the broken stick model applied to hydro-meteorical data provided an underestimate of the dimensionality of the data. Her claim is based on the fact that other heuristic techniques generally gave higher numbers (2 versus 5 to 6). However, it should be noted that the true dimension of the data is unknown. Ferré [21] suggests that since PCA is used primarily for descriptive rather than predictive purposes, which has been the case with microarray data analysis, any solution less than the true dimension is acceptable.

The broken-stick model has the advantage of being extremely easy to calculate and implement. Consider the closed interval *J *= [0,1]. Suppose *J *is partitioned into *n *subintervals by randomly selecting *n *- 1 points from a uniform distribution in the same interval. Arrange the subintervals according to length in descending order and denote by *L*_*k *_the length of the *k*-th subinterval. Then the expected value of *L*_*k *_is [37]

E(Lk)=1n∑j=kn1j.     (5)
 MathType@MTEF@5@5@+=feaafiart1ev1aaatCvAUfKttLearuWrP9MDH5MBPbIqV92AaeXatLxBI9gBaebbnrfifHhDYfgasaacH8akY=wiFfYdH8Gipec8Eeeu0xXdbba9frFj0=OqFfea0dXdd9vqai=hGuQ8kuc9pgc9s8qqaq=dirpe0xb9q8qiLsFr0=vr0=vr0dc8meaabaqaciaacaGaaeqabaqabeGadaaakeaacqWGfbqrdaqadaqaaiabdYeamnaaBaaaleaacqWGRbWAaeqaaaGccaGLOaGaayzkaaGaeyypa0ZaaSaaaeaacqaIXaqmaeaacqWGUbGBaaWaaabCaeaadaWcaaqaaiabigdaXaqaaiabdQgaQbaacqGGUaGlaSqaaiabdQgaQjabg2da9iabdUgaRbqaaiabd6gaUbqdcqGHris5aOGaaCzcaiaaxMaadaqadaqaaiabiwda1aGaayjkaiaawMcaaaaa@43E2@

**Figure 2:**
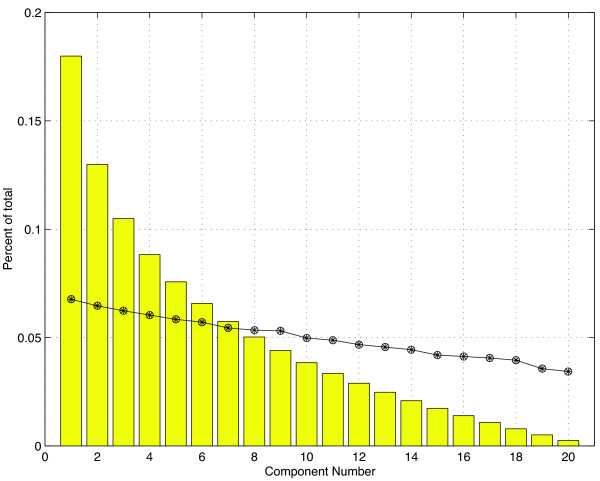
**The broken stick method**. The broken stick distribution (bars) with eigenvalues obtained from a uniform random matrix of size 500 × 20.

Figure 2 provides an illustration of the broken stick distribution for *n *= 20 subintervals graphed along with eigenvalues obtained from the covariance matrix of a random matrix. The elements of the random matrix are drawn from a uniform distribution on the interval [0,1]. The bars represent the values from the broken stick distribution; the circles represent the eigenvalues of the random matrix. In this case, no component would be retained since the proportion of variance explained by the first (largest) eigenvalue falls below the first value given by the broken stick model.

### 2.3 Modified broken stick model

Consider a subspace spanned by eigenvectors associated with a set of "nearly equal" eigenvalues that are "well separated" from all other eigenvalues. Such a subspace is well defined in that it is orthogonal to the subspaces spanned by the remaining eigenvectors; however, individual principal components within that subspace are unstable [9]. This instability is described in North *et al. *[38] where a first order approximation to estimate how sample eigenvalues and eigenvectors differ from their exact quantities is derived. This "rule of thumb" estimate is

*δλ *~ *λ *(2/*N*)^1/2 ^    (6)

where *N *is the sample size and *λ *is an eigenvalue. The interpretation given by North *et al*. [38] is that "... if a group of true eigenvalues lie within one or two *δλ *of each other then they form an 'effectively degenerate multiplex,' and sample eigenvectors are a random mixture of the true eigenvectors."

As noted previously, the broken stick model has been referred to as a resource apportionment model, and in particular, the resource to be apportioned among the components is the total variance. We modify this approach by considering the variance as apportioned among individual subspaces.

Once the eigenvalues, *λ*_*i*_, have been computed, the spacing between them, *λ*_*i*+1 _- *λ*_*i*_, is calculated. Using Equation (6), an estimate of the sampling error is determined and those eigenvalues which lie within 1.5 of each other is noted (note that the value of the spacing, 1.5, is somewhat arbitrary. In their report, North *et al. *[38] suggest using a value between 1 and 2.) Components are then grouped into subspaces preserving the order determined by the maximum variance property of PCA. Subspaces are spanned by either a single eigenvector, or in the case of an "effective degeneracy," by multiple eigenvectors. Denote these subspaces by *W*_*i*_. For each *W*_*i *_we sum the eigenvalues associated with the eigenvectors spanning that space. We then repartition the broken stick model to match the subspaces and then apply the broken stick model to each subspace, requiring that the sum of the eigenvalues associated with that subspace exceed the value given by the broken stick model.

### 2.4 Statistical entropy and dimensionality

In physics, the entropy of a closed system is a measure of disorder. An increase in entropy corresponds to an increase in disorder which is accompanied by a decrease in information. In the branch of applied probability known as information theory [39-42], the concept of entropy is used to provide a measure of the uncertainty or information of a system. Note that uncertainty and information are used synonymously, the reason for which is explained below. The word system is used to imply a complete discrete random experiment.

#### 2.4.1 Information and uncertainty

In the context of information theory, information is a measure of what can be communicated rather than what is communicated [42]. Information can also be though of as a term used to describe a process that selects one or more objects from a set of object. For example, consider a balanced six-sided die. We will consider the die as a device, *D*, that can produce with equal probability any element of the set *S*_*D *_= {1, 2, 3,4, 5, 6}. Of course, observing a tossed die and noting the number of the top face is a finite discrete probability experiment with sample space *S*_*D *_where each sample point is equally likely to occur.

Denote the probability model for an experiment with outcomes *e*_1_,...,*e*_*n *_with associated probabilities *p*_1_,...,*p*_*n *_as

(e1e2⋯enp1p2⋯pn).     (7)
 MathType@MTEF@5@5@+=feaafiart1ev1aaatCvAUfKttLearuWrP9MDH5MBPbIqV92AaeXatLxBI9gBaebbnrfifHhDYfgasaacH8akY=wiFfYdH8Gipec8Eeeu0xXdbba9frFj0=OqFfea0dXdd9vqai=hGuQ8kuc9pgc9s8qqaq=dirpe0xb9q8qiLsFr0=vr0=vr0dc8meaabaqaciaacaGaaeqabaqabeGadaaakeaadaqadaqaauaabaqacqaaaaqaaiabdwgaLnaaBaaaleaacqaIXaqmaeqaaaGcbaGaemyzau2aaSbaaSqaaiabikdaYaqabaaakeaacqWIVlctaeaacqWGLbqzdaWgaaWcbaGaemOBa4gabeaaaOqaaiabdchaWnaaBaaaleaacqaIXaqmaeqaaaGcbaGaemiCaa3aaSbaaSqaaiabikdaYaqabaaakeaacqWIVlctaeaacqWGWbaCdaWgaaWcbaGaemOBa4gabeaaaaaakiaawIcacaGLPaaacqGGUaGlcaWLjaGaaCzcamaabmaabaGaeG4naCdacaGLOaGaayzkaaaaaa@46D9@

In the example of the balanced six-sided die we have

(1234561/61/61/61/61/61/6).     (8)
 MathType@MTEF@5@5@+=feaafiart1ev1aaatCvAUfKttLearuWrP9MDH5MBPbIqV92AaeXatLxBI9gBaebbnrfifHhDYfgasaacH8akY=wiFfYdH8Gipec8Eeeu0xXdbba9frFj0=OqFfea0dXdd9vqai=hGuQ8kuc9pgc9s8qqaq=dirpe0xb9q8qiLsFr0=vr0=vr0dc8meaabaqaciaacaGaaeqabaqabeGadaaakeaadaqadaqaauaabeqacyaaaaqaaiabigdaXaqaaiabikdaYaqaaiabiodaZaqaaiabisda0aqaaiabiwda1aqaaiabiAda2aqaaiabigdaXiabc+caViabiAda2aqaaiabigdaXiabc+caViabiAda2aqaaiabigdaXiabc+caViabiAda2aqaaiabigdaXiabc+caViabiAda2aqaaiabigdaXiabc+caViabiAda2aqaaiabigdaXiabc+caViabiAda2aaaaiaawIcacaGLPaaacqGGUaGlcaWLjaGaaCzcamaabmaabaGaeGioaGdacaGLOaGaayzkaaaaaa@499E@

Prior to performing the experiment, we are uncertain as to its outcome. Once the die is tossed and we receive information regarding the outcome, the uncertainty decreases. As a measure of this uncertainty we can say that the device has an "uncertainty of six symbols" [43]. Now consider a "fair" coin. This "device," which we will call C, produces two symbols with equal likelihood from the set *S*_*C *_= {*h*, *t*} and we say this device has an "uncertainty of two symbols." We denote this probability model as

(ht1/21/2).     (9)
 MathType@MTEF@5@5@+=feaafiart1ev1aaatCvAUfKttLearuWrP9MDH5MBPbIqV92AaeXatLxBI9gBaebbnrfifHhDYfgasaacH8akY=wiFfYdH8Gipec8Eeeu0xXdbba9frFj0=OqFfea0dXdd9vqai=hGuQ8kuc9pgc9s8qqaq=dirpe0xb9q8qiLsFr0=vr0=vr0dc8meaabaqaciaacaGaaeqabaqabeGadaaakeaadaqadaqaauaabeqaciaaaeaacqWGObaAaeaacqWG0baDaeaacqaIXaqmcqGGVaWlcqaIYaGmaeaacqaIXaqmcqGGVaWlcqaIYaGmaaaacaGLOaGaayzkaaGaeiOla4IaaCzcaiaaxMaadaqadaqaaiabiMda5aGaayjkaiaawMcaaaaa@3B50@

Both models represent uniform distributions (the outcomes in the respective models have equal probabilities), but it is inferred that device *D *is a finite scheme with greater uncertainty than device *C *(an "uncertainty of six symbols" versus an "uncertainty of two symbols"). Consequently, we expect device *D *to convey more information. To see this, consider (as an approximation to the amount of information conveyed) the average minimum number of binary questions that would be required to ascertain the outcome of each experiment. In the case of device *D*, the average minimum number of questions is 2.4 while in the case of device *C *only one question is required. Now consider an oddly minted coin with identical sides (say heads on either side). The model for this device is

(ht10).     (10)
 MathType@MTEF@5@5@+=feaafiart1ev1aaatCvAUfKttLearuWrP9MDH5MBPbIqV92AaeXatLxBI9gBaebbnrfifHhDYfgasaacH8akY=wiFfYdH8Gipec8Eeeu0xXdbba9frFj0=OqFfea0dXdd9vqai=hGuQ8kuc9pgc9s8qqaq=dirpe0xb9q8qiLsFr0=vr0=vr0dc8meaabaqaciaacaGaaeqabaqabeGadaaakeaadaqadaqaauaabeqaciaaaeaacqWGObaAaeaacqWG0baDaeaacqaIXaqmaeaacqaIWaamaaaacaGLOaGaayzkaaGaeiOla4IaaCzcaiaaxMaadaqadaqaaiabigdaXiabicdaWaGaayjkaiaawMcaaaaa@387C@

Since heads, *h*, is the only possible outcome, we consider this as a "device of one symbol." Notice that this device carries no information and contains no element of uncertainty. We need not pose a question to ascertain the outcome of the experiment. Thus, a function that attempts to quantify the information or uncertainty of a system will depend on the cardinality of the sample space and the probability distribution.

#### 2.4.2 Entropy: a measure of information content (or uncertainty)

Every probability model (or device) describes a state of uncertainty [41]. Shannon [42] provided a measure for such uncertainty, which is known as statistical entropy (often referred to as Shannon's entropy). Its functional form is given by

H(p1,...,pN)=−∑k=1Npklog⁡2pk.     (11)
 MathType@MTEF@5@5@+=feaafiart1ev1aaatCvAUfKttLearuWrP9MDH5MBPbIqV92AaeXatLxBI9gBaebbnrfifHhDYfgasaacH8akY=wiFfYdH8Gipec8Eeeu0xXdbba9frFj0=OqFfea0dXdd9vqai=hGuQ8kuc9pgc9s8qqaq=dirpe0xb9q8qiLsFr0=vr0=vr0dc8meaabaqaciaacaGaaeqabaqabeGadaaakeaacqWGibasdaqadaqaaiabdchaWnaaBaaaleaacqaIXaqmaeqaaOGaeiilaWIaeiOla4IaeiOla4IaeiOla4IaeiilaWIaemiCaa3aaSbaaSqaaiabd6eaobqabaaakiaawIcacaGLPaaacqGH9aqpcqGHsisldaaeWbqaaiabdchaWnaaBaaaleaacqWGRbWAaeqaaOGagiiBaWMaei4Ba8Maei4zaC2aaSbaaSqaaiabikdaYaqabaGccqWGWbaCdaWgaaWcbaGaem4AaSgabeaakiabc6caUiaaxMaacaWLjaWaaeWaaeaacqaIXaqmcqaIXaqmaiaawIcacaGLPaaaaSqaaiabdUgaRjabg2da9iabigdaXaqaaiabd6eaobqdcqGHris5aaaa@528F@

Equation (11) represents the average information content or average uncertainty of a discrete system. The quantity is considered a measure of information or uncertainty depending upon whether we consider ourselves in the moment before the experiment (uncertainty) or in a moment after the experiment (information), [54].

#### 2.4.3 Derivation of H

Statistical entropy is derived from the negative binomial distribution where an experiment with two equally likely outcomes, labeled success or failure, is considered. Basilevsky [26] shows that with *x *representing the first number on which a "success" is observed, the probability of observing success on the *x*^*th *^trial is given by *f*(*x*) = *p *= (l/2)^*x*^. Upon solving for *x *we have *x *= -log_2 _*p*, and the expected or total entropy of a system, *H*, is

H=−∑xf(x)x=∑k=1Npklog⁡21pk,     (12)
 MathType@MTEF@5@5@+=feaafiart1ev1aaatCvAUfKttLearuWrP9MDH5MBPbIqV92AaeXatLxBI9gBaebbnrfifHhDYfgasaacH8akY=wiFfYdH8Gipec8Eeeu0xXdbba9frFj0=OqFfea0dXdd9vqai=hGuQ8kuc9pgc9s8qqaq=dirpe0xb9q8qiLsFr0=vr0=vr0dc8meaabaqaciaacaGaaeqabaqabeGadaaakeaacqWGibascqGH9aqpcqGHsisldaaeqbqaaiabdAgaMnaabmaabaGaemiEaGhacaGLOaGaayzkaaGaemiEaGNaeyypa0ZaaabCaeaacqWGWbaCdaWgaaWcbaGaem4AaSgabeaakiGbcYgaSjabc+gaVjabcEgaNnaaBaaaleaacqaIYaGmaeqaaOWaaSaaaeaacqaIXaqmaeaacqWGWbaCdaWgaaWcbaGaem4AaSgabeaaaaGccqGGSaalcaWLjaGaaCzcamaabmaabaGaeGymaeJaeGOmaidacaGLOaGaayzkaaaaleaacqWGRbWAcqGH9aqpcqaIXaqmaeaacqWGobGta0GaeyyeIuoaaSqaaiabdIha4bqab0GaeyyeIuoaaaa@52B7@

where *p*_*k *_log_2 _1pk
 MathType@MTEF@5@5@+=feaafiart1ev1aaatCvAUfKttLearuWrP9MDH5MBPbIqV92AaeXatLxBI9gBaebbnrfifHhDYfgasaacH8akY=wiFfYdH8Gipec8Eeeu0xXdbba9frFj0=OqFfea0dXdd9vqai=hGuQ8kuc9pgc9s8qqaq=dirpe0xb9q8qiLsFr0=vr0=vr0dc8meaabaqaciaacaGaaeqabaqabeGadaaakeaadaWcaaqaaiabigdaXaqaaiabdchaWnaaBaaaleaacqWGRbWAaeqaaaaaaaa@30A0@ is defined to be 0 if *p*_*k *_= 0.

#### 2.4.4 Basic properties

It is possible to derive the form of the function *H *by assuming it possesses four basic properties, [41]: (i) continuity, (ii) symmetry, (iii) an extremal property, and (iv) additivity. Continuity requires that the measure of uncertainty varies continuously if the probabilities of the outcomes of an experiment are varied in a continuous way. Symmetry states that the measure must be invariant to the order of the p′k
 MathType@MTEF@5@5@+=feaafiart1ev1aaatCvAUfKttLearuWrP9MDH5MBPbIqV92AaeXatLxBI9gBaebbnrfifHhDYfgasaacH8akY=wiFfYdH8Gipec8Eeeu0xXdbba9frFj0=OqFfea0dXdd9vqai=hGuQ8kuc9pgc9s8qqaq=dirpe0xb9q8qiLsFr0=vr0=vr0dc8meaabaqaciaacaGaaeqabaqabeGadaaakeaacuWGWbaCgaqbamaaBaaaleaacqWGRbWAaeqaaaaa@2FAC@*s*, that is, *H*(*p*_1_, *p*_2_,...,*p*_*N*_) = *H*(*p*_2_, *p*_1_,...,*p*_*N*_). Additivity requires that given the following three *H *functions defined on the same probability space

*H*_1_(*p*_1_, *p*_2_,...,*p*_*N*_),

*H*_2_(*p*_1_, *p*_2_,...,*p*_*N*_, *q*_1_, *q*_2_,...,*q*_*M*_),     (13)

*H*_3_(*q*_1_/*p*_*N*_, *q*_2_/*p*_*N*_,...,*q*_*M*_/*p*_*N*_),

the relationship *H*_2 _= *H*_1 _+ *p*_*N*_*H*_3 _holds. Notice that this implies *H*_2 _≥ *H*_3_, that is, partitioning events into sub-events cannot decrease the entropy of the system [39]. The extremal property, which we now describe, will be used in our development of the information dimension described below. First, notice that since 0 ≤ *p*_*k *_≤ 1 for all *k*, a minimum value of 0 is attained when *p*_1 _= 1 and *p*_2 _= *p*_3 _= ⋯ = *p*_*N *_= 0, so *H *≥ 0. As an upper bound, we have

H(p1,p2,...,pN)≤H(1N,1N,...,1N)=log⁡2N,     (14)
 MathType@MTEF@5@5@+=feaafiart1ev1aaatCvAUfKttLearuWrP9MDH5MBPbIqV92AaeXatLxBI9gBaebbnrfifHhDYfgasaacH8akY=wiFfYdH8Gipec8Eeeu0xXdbba9frFj0=OqFfea0dXdd9vqai=hGuQ8kuc9pgc9s8qqaq=dirpe0xb9q8qiLsFr0=vr0=vr0dc8meaabaqaciaacaGaaeqabaqabeGadaaakeaacqWGibasdaqadaqaaiabdchaWnaaBaaaleaacqaIXaqmaeqaaOGaeiilaWIaemiCaa3aaSbaaSqaaiabikdaYaqabaGccqGGSaalcqGGUaGlcqGGUaGlcqGGUaGlcqGGSaalcqWGWbaCdaWgaaWcbaGaemOta4eabeaaaOGaayjkaiaawMcaaiabgsMiJkabdIeainaabmaabaWaaSaaaeaacqaIXaqmaeaacqWGobGtaaGaeiilaWYaaSaaaeaacqaIXaqmaeaacqWGobGtaaGaeiilaWIaeiOla4IaeiOla4IaeiOla4IaeiilaWYaaSaaaeaacqaIXaqmaeaacqWGobGtaaaacaGLOaGaayzkaaGaeyypa0JagiiBaWMaei4Ba8Maei4zaC2aaSbaaSqaaiabikdaYaqabaGccqWGobGtcqGGSaalcaWLjaGaaCzcamaabmaabaGaeGymaeJaeGinaqdacaGLOaGaayzkaaaaaa@5994@

that is, a uniform distribution of probabilities provides an upper bound on the uncertainty measure of all discrete probability models whose sample space has cardinality of at most *N*. This relationship can be proved either with differential calculus [39] or from Jensen's inequality

ϕ(1N∑k=1Nak)≤1N∑k=1Nϕ(ak)     (15)
 MathType@MTEF@5@5@+=feaafiart1ev1aaatCvAUfKttLearuWrP9MDH5MBPbIqV92AaeXatLxBI9gBaebbnrfifHhDYfgasaacH8akY=wiFfYdH8Gipec8Eeeu0xXdbba9frFj0=OqFfea0dXdd9vqai=hGuQ8kuc9pgc9s8qqaq=dirpe0xb9q8qiLsFr0=vr0=vr0dc8meaabaqaciaacaGaaeqabaqabeGadaaakeaaiiGacqWFvpGAdaqadaqaamaalaaabaGaeGymaedabaGaemOta4eaamaaqahabaGaemyyae2aaSbaaSqaaiabdUgaRbqabaaabaGaem4AaSMaeyypa0JaeGymaedabaGaemOta4eaniabggHiLdaakiaawIcacaGLPaaacqGHKjYOdaWcaaqaaiabigdaXaqaaiabd6eaobaadaaeWbqaaiab=v9aQnaabmaabaGaemyyae2aaSbaaSqaaiabdUgaRbqabaaakiaawIcacaGLPaaacaWLjaGaaCzcamaabmaabaGaeGymaeJaeGynaudacaGLOaGaayzkaaaaleaacqWGRbWAcqGH9aqpcqaIXaqmaeaacqWGobGta0GaeyyeIuoaaaa@5139@

which is valid for any convex function *ϕ *[41].

#### 2.4.5 The information dimension, n_0_

What we coin as, the "information dimension," *n*_0_, is presented as a novel (but heuristic) measure for the interpretable components in a principal component analysis. We assume that a principal component analysis has been performed on a microarray data set and that our objective is to reduce the dimension of the data by retaining "meaningful" components. This involves setting one or more of the eigenvalues associated with the low variance components to zero. Let *λ*_1_, *λ*_2_,...,*λ*_*N *_represent the eigenvalues from a PCA of the data. Define for each *k*

pk=λk∑j=1Nλj.     (16)
 MathType@MTEF@5@5@+=feaafiart1ev1aaatCvAUfKttLearuWrP9MDH5MBPbIqV92AaeXatLxBI9gBaebbnrfifHhDYfgasaacH8akY=wiFfYdH8Gipec8Eeeu0xXdbba9frFj0=OqFfea0dXdd9vqai=hGuQ8kuc9pgc9s8qqaq=dirpe0xb9q8qiLsFr0=vr0=vr0dc8meaabaqaciaacaGaaeqabaqabeGadaaakeaacqWGWbaCdaWgaaWcbaGaem4AaSgabeaakiabg2da9maalaaabaacciGae83UdW2aaSbaaSqaaiabdUgaRbqabaaakeaadaaeWaqaaiab=T7aSnaaBaaaleaacqWGQbGAaeqaaaqaaiabdQgaQjabg2da9iabigdaXaqaaiabd6eaobqdcqGHris5aaaakiabc6caUiaaxMaacaWLjaWaaeWaaeaacqaIXaqmcqaI2aGnaiaawIcacaGLPaaaaaa@435C@

The p′k
 MathType@MTEF@5@5@+=feaafiart1ev1aaatCvAUfKttLearuWrP9MDH5MBPbIqV92AaeXatLxBI9gBaebbnrfifHhDYfgasaacH8akY=wiFfYdH8Gipec8Eeeu0xXdbba9frFj0=OqFfea0dXdd9vqai=hGuQ8kuc9pgc9s8qqaq=dirpe0xb9q8qiLsFr0=vr0=vr0dc8meaabaqaciaacaGaaeqabaqabeGadaaakeaacuWGWbaCgaqbamaaBaaaleaacqWGRbWAaeqaaaaa@2FAC@*s *satisfy 0 ≤ *p*_*k *_≤ 1, (*k *= 1,...,*N*) and ∑K=1Npk=1
 MathType@MTEF@5@5@+=feaafiart1ev1aaatCvAUfKttLearuWrP9MDH5MBPbIqV92AaeXatLxBI9gBaebbnrfifHhDYfgasaacH8akY=wiFfYdH8Gipec8Eeeu0xXdbba9frFj0=OqFfea0dXdd9vqai=hGuQ8kuc9pgc9s8qqaq=dirpe0xb9q8qiLsFr0=vr0=vr0dc8meaabaqaciaacaGaaeqabaqabeGadaaakeaadaaeWaqaaiabdchaWnaaBaaaleaacqWGRbWAaeqaaOGaeyypa0JaeGymaedaleaacqWGlbWscqGH9aqpcqaIXaqmaeaacqWGobGta0GaeyyeIuoaaaa@37DC@. We view the distribution of the eigenvalues expressed as a proportion of total variance as a discrete probability model.

We begin by normalizing the entropy measure [10] for some fixed *n *= *N *using its extremal property to get

H˜=Hlog⁡2(N)=−1log⁡2(N)∑k=1Npklog⁡2pk.     (17)
 MathType@MTEF@5@5@+=feaafiart1ev1aaatCvAUfKttLearuWrP9MDH5MBPbIqV92AaeXatLxBI9gBaebbnrfifHhDYfgasaacH8akY=wiFfYdH8Gipec8Eeeu0xXdbba9frFj0=OqFfea0dXdd9vqai=hGuQ8kuc9pgc9s8qqaq=dirpe0xb9q8qiLsFr0=vr0=vr0dc8meaabaqaciaacaGaaeqabaqabeGadaaakeaadaaiaaqaaiabdIeaibGaay5adaGaeyypa0ZaaSaaaeaacqWGibasaeaacyGGSbaBcqGGVbWBcqGGNbWzdaWgaaWcbaGaeGOmaidabeaakmaabmaabaGaemOta4eacaGLOaGaayzkaaaaaiabg2da9iabgkHiTmaalaaabaGaeGymaedabaGagiiBaWMaei4Ba8Maei4zaC2aaSbaaSqaaiabikdaYaqabaGcdaqadaqaaiabd6eaobGaayjkaiaawMcaaaaadaaeWbqaaiabdchaWnaaBaaaleaacqWGRbWAaeqaaOGagiiBaWMaei4Ba8Maei4zaC2aaSbaaSqaaiabikdaYaqabaGccqWGWbaCdaWgaaWcbaGaem4AaSgabeaakiabc6caUiaaxMaacaWLjaWaaeWaaeaacqaIXaqmcqaI3aWnaiaawIcacaGLPaaaaSqaaiabdUgaRjabg2da9iabigdaXaqaaiabd6eaobqdcqGHris5aaaa@5B2C@

The values of H˜
 MathType@MTEF@5@5@+=feaafiart1ev1aaatCvAUfKttLearuWrP9MDH5MBPbIqV92AaeXatLxBI9gBaebbnrfifHhDYfgasaacH8akY=wiFfYdH8Gipec8Eeeu0xXdbba9frFj0=OqFfea0dXdd9vqai=hGuQ8kuc9pgc9s8qqaq=dirpe0xb9q8qiLsFr0=vr0=vr0dc8meaabaqaciaacaGaaeqabaqabeGadaaakeaacuWGibasgaacaaaa@2DD4@ will vary between 0 and 1 inclusive.

We calculate the entropy of the probability space using Equation (17) to obtain the functional value H˜
 MathType@MTEF@5@5@+=feaafiart1ev1aaatCvAUfKttLearuWrP9MDH5MBPbIqV92AaeXatLxBI9gBaebbnrfifHhDYfgasaacH8akY=wiFfYdH8Gipec8Eeeu0xXdbba9frFj0=OqFfea0dXdd9vqai=hGuQ8kuc9pgc9s8qqaq=dirpe0xb9q8qiLsFr0=vr0=vr0dc8meaabaqaciaacaGaaeqabaqabeGadaaakeaacuWGibasgaacaaaa@2DD4@, where 0 ≤ H˜
 MathType@MTEF@5@5@+=feaafiart1ev1aaatCvAUfKttLearuWrP9MDH5MBPbIqV92AaeXatLxBI9gBaebbnrfifHhDYfgasaacH8akY=wiFfYdH8Gipec8Eeeu0xXdbba9frFj0=OqFfea0dXdd9vqai=hGuQ8kuc9pgc9s8qqaq=dirpe0xb9q8qiLsFr0=vr0=vr0dc8meaabaqaciaacaGaaeqabaqabeGadaaakeaacuWGibasgaacaaaa@2DD4@ ≤ 1. (Note that at either extreme the dimension is known.) Next, we deform the original distribution of eigenvalues so that the following holds

p1=⋯=pn0=1n0     (18)
 MathType@MTEF@5@5@+=feaafiart1ev1aaatCvAUfKttLearuWrP9MDH5MBPbIqV92AaeXatLxBI9gBaebbnrfifHhDYfgasaacH8akY=wiFfYdH8Gipec8Eeeu0xXdbba9frFj0=OqFfea0dXdd9vqai=hGuQ8kuc9pgc9s8qqaq=dirpe0xb9q8qiLsFr0=vr0=vr0dc8meaabaqaciaacaGaaeqabaqabeGadaaakeaacqWGWbaCdaWgaaWcbaGaeGymaedabeaakiabg2da9iabl+Uimjabg2da9iabdchaWnaaBaaaleaacqWGUbGBdaWgaaadbaGaeGimaadabeaaaSqabaGccqGH9aqpdaWcaaqaaiabigdaXaqaaiabd6gaUnaaBaaaleaacqaIWaamaeqaaaaakiaaxMaacaWLjaWaaeWaaeaacqaIXaqmcqaI4aaoaiaawIcacaGLPaaaaaa@40A9@

pn0+1=⋯=pN=0,     (19)
 MathType@MTEF@5@5@+=feaafiart1ev1aaatCvAUfKttLearuWrP9MDH5MBPbIqV92AaeXatLxBI9gBaebbnrfifHhDYfgasaacH8akY=wiFfYdH8Gipec8Eeeu0xXdbba9frFj0=OqFfea0dXdd9vqai=hGuQ8kuc9pgc9s8qqaq=dirpe0xb9q8qiLsFr0=vr0=vr0dc8meaabaqaciaacaGaaeqabaqabeGadaaakeaacqWGWbaCdaWgaaWcbaGaemOBa42aaSbaaWqaaiabicdaWaqabaWccqGHRaWkcqaIXaqmaeqaaOGaeyypa0JaeS47IWKaeyypa0JaemiCaa3aaSbaaSqaaiabd6eaobqabaGccqGH9aqpcqaIWaamcqGGSaalcaWLjaGaaCzcamaabmaabaGaeGymaeJaeGyoaKdacaGLOaGaayzkaaaaaa@40F7@

Inserting these values into H˜
 MathType@MTEF@5@5@+=feaafiart1ev1aaatCvAUfKttLearuWrP9MDH5MBPbIqV92AaeXatLxBI9gBaebbnrfifHhDYfgasaacH8akY=wiFfYdH8Gipec8Eeeu0xXdbba9frFj0=OqFfea0dXdd9vqai=hGuQ8kuc9pgc9s8qqaq=dirpe0xb9q8qiLsFr0=vr0=vr0dc8meaabaqaciaacaGaaeqabaqabeGadaaakeaacuWGibasgaacaaaa@2DD4@ and solving for *n*_0 _yields

n0=NH˜0=∏k=1Npk−pk.     (20)
 MathType@MTEF@5@5@+=feaafiart1ev1aaatCvAUfKttLearuWrP9MDH5MBPbIqV92AaeXatLxBI9gBaebbnrfifHhDYfgasaacH8akY=wiFfYdH8Gipec8Eeeu0xXdbba9frFj0=OqFfea0dXdd9vqai=hGuQ8kuc9pgc9s8qqaq=dirpe0xb9q8qiLsFr0=vr0=vr0dc8meaabaqaciaacaGaaeqabaqabeGadaaakeaacqWGUbGBdaWgaaWcbaGaeGimaadabeaakiabg2da9iabd6eaonaaCaaaleqabaWaaacaaeaacqWGibasaiaawoWaamaaBaaameaacqaIWaamaeqaaaaakiabg2da9maarahabaGaemiCaa3aa0baaSqaaiabdUgaRbqaaiabgkHiTiabdchaWnaaBaaameaacqWGRbWAaeqaaaaaaSqaaiabdUgaRjabg2da9iabigdaXaqaaiabd6eaobqdcqGHpis1aOGaeiOla4IaaCzcaiaaxMaadaqadaqaaiabikdaYiabicdaWaGaayjkaiaawMcaaaaa@48B0@

#### 2.4.6 A geometric example

Consider the following geometric example. The surface of a three-dimensional ellipsoid is parameterized by the equations

*x*(*φ*,*θ*) = *R*_*x*_sin(*φ*)cos(*θ*),

*y*(*φ*,*θ*) = *R*_*y*_sin(*φ*)sin(*θ*),     (21)

*z*(*φ*,*θ*) = *R*_*z*_cos(*φ*).

Points are distributed along the surface of the ellipsoid according to the above parametrization and are tabulated in the matrix *X *of size (4584 × 3). Set *R*_*x *_= *R*_*y *_= *R*_*z *_= 1, then (21) is a parametrization representing the surface of the unit sphere centered at the origin. Gradually deform the sphere by changing the values of *R*_*i *_subject to the constraint *R*_*x*_*R*_*y*_*R*_*z *_= 1, which gives ellipsoids of constant volume (equal to 4*π*/3). We summarize the results in Table 1.

**Table 1:** Dimension suggested by the Information Dimension

(*R*_*x*_, *R*_*y*_, *R*_*z*_)	(1,1,1)	(8/5,1,5/8)	(2,1,1/2)	(3,1,1/3)	(8,1,1/8)
*H*(*p*_1_,*p*_2_,*p*_3_)	1.000	0.781	0.608	0.348	0.074
*p*_1 _= *λ*_1_/(*λ*_1 _+ *λ*_2 _+ *λ*_3_)	0.333	0.648	0.762	0.890	0.984
*p*_2 _= *λ*_2_/(*λ*_1 _+ *λ*_2 _+ *λ*_3_)	0.333	0.253	0.191	0.099	0.015
*p*_3 _= *λ*_3_/(*λ*_1 _+ *λ*_2 _+ *λ*_3_)	0.333	0.099	0.048	0.010	0.000
*n*_0_	3.00	2.36	1.95	1.47	1.09

Notice that for the case *R*_*x *_= *R*_*y *_= *R*_*z *_= 1, which represents the unit sphere, *n*_0 _= 3. The gradual deformation of the sphere has an information dimension of approximately two for the values: *R*_*x *_= 2, *R*_*y *_= 1, *R*_*z *_= 1/2. This suggests that the magnitude along the *z*-axis has become sufficiently small relative to the *x*- and *y*-axes, that it may be discarded for information purposes. Thus, a projection onto the *xy*-plane may provide sufficient information regarding the shape of the object. For *R*_*x *_= 8, *R*_*y *_= 1, *R*_*z *_= 1/8 the object begins to "look" one dimensional with *n*_0 _= 1.09. With this configuration, most of the variance lies along the *x*-axis.

## 3 Results and discussion

In this section we apply the information dimension, the broken stick model, the modified broken stick model, Bartlett's Test, Kaiser-Guttman, Jolliffe's modification of Kaiser-Guttman, Velicer's minimum average partial (MAP) criteria, Cattell's scree test, parallel analysis, cumulative percent of variance explained, and Log-eigenvalue diagram techniques to published yeast cdc15 cell-cycle and elutriation-synchronized cell cycle data sets [34], sporulation data set [44], serum-treated human fibroblast data set [45], and the cancer cell lines data sets [46]. These data sets have been previously explored [10-12,47].

Before attempting to reduce the dimension of the data, we first consider whether a PCA is appropriate, that is, a data set with very high information content will not lend itself to significant dimension reduction, at least not without some non-trivial loss of information. In their study, Alter *et al. *[10] addresses the issue by considering the normalized entropy (presented above) of a data set, which is a measure of the complexity or redundancy of the data. The index ranges in value from 0 to 1 with values near zero indicating low information content versus values near 1 which indicate a highly disordered or random data set. In this form, the entropy can only be use to give the researcher a "feeling" for the potential for dimension reduction. For example, what level of dimension reduction is implied by an entropy reading of 0.3 versus 0.4?

Another measure is presented in Jackson [7] and credited to Gleason and Staelin for use with the *q *× *q *correlation matrix, *R*, and is given by

ϑ=‖R‖2−qq(q−1),     (22)
 MathType@MTEF@5@5@+=feaafiart1ev1aaatCvAUfKttLearuWrP9MDH5MBPbIqV92AaeXatLxBI9gBaebbnrfifHhDYfgasaacH8akY=wiFfYdH8Gipec8Eeeu0xXdbba9frFj0=OqFfea0dXdd9vqai=hGuQ8kuc9pgc9s8qqaq=dirpe0xb9q8qiLsFr0=vr0=vr0dc8meaabaqaciaacaGaaeqabaqabeGadaaakeaacqaHrpGscqGH9aqpdaGcaaqaamaalaaabaWaauWaaeaacqWGsbGuaiaawMa7caGLkWoadaahaaWcbeqaaiabikdaYaaakiabgkHiTiabdghaXbqaaiabdghaXnaabmaabaGaemyCaeNaeyOeI0IaeGymaedacaGLOaGaayzkaaaaaaWcbeaakiabcYcaSiaaxMaacaWLjaWaaeWaaeaacqaIYaGmcqaIYaGmaiaawIcacaGLPaaaaaa@4331@

The statistic also ranges in value from 0 to 1. If there is little or no correlation among the variables, the statistic will be close to 0; a set of highly correlated variables will have a statistic close to 1. The statistic is given by Jackson [7] asserts that the distribution of the statistic is unknown, but may be useful in comparing data sets.

### 3.1 Stopping rules applied to synthetic data

In this section we apply the stopping criteria to a (6000 × 15) matrix, *X*_*σ*_, where *X*_*σ *_is populated with simulated data. The simulation model can be expressed as *X*_*σ *_= *Y *+ *N*_*σ*_, where *N*_*σ *_is a random matrix representing Gaussian noise and whose entries were drawn from a standard normal distribution with zero mean and standard deviation, *σ*. The matrix *Y *was constructed by populating the first 600 rows with values from six orthonormal polynomials. Each polynomial populates 100 rows of the matrix. The polynomials were constructed using a Gramm-Schmidt process [48] with norm

**Figure 3:**
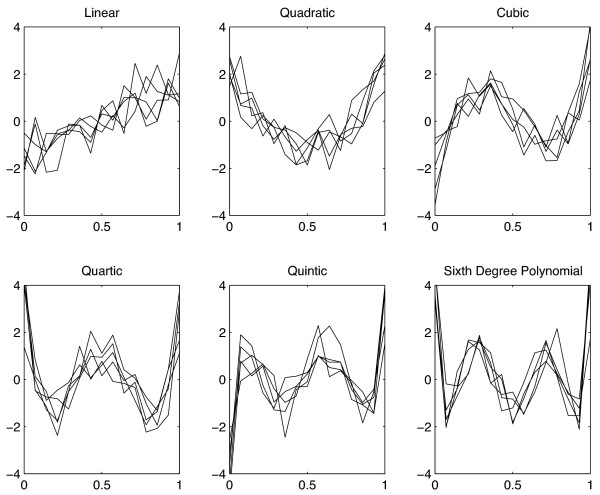
**Simulated data**. Six orthonormal polynomials with gaussian Noise.

∫01pj(x)qk(x)dx=δjk,     (23)
 MathType@MTEF@5@5@+=feaafiart1ev1aaatCvAUfKttLearuWrP9MDH5MBPbIqV92AaeXatLxBI9gBaebbnrfifHhDYfgasaacH8akY=wiFfYdH8Gipec8Eeeu0xXdbba9frFj0=OqFfea0dXdd9vqai=hGuQ8kuc9pgc9s8qqaq=dirpe0xb9q8qiLsFr0=vr0=vr0dc8meaabaqaciaacaGaaeqabaqabeGadaaakeaadaWdXaqaaiabdchaWnaaBaaaleaacqWGQbGAaeqaaOWaaeWaaeaacqWG4baEaiaawIcacaGLPaaacqWGXbqCdaWgaaWcbaGaem4AaSgabeaakmaabmaabaGaemiEaGhacaGLOaGaayzkaaGaemizaqMaemiEaGNaeyypa0dcciGae8hTdq2aaSbaaSqaaiabdQgaQjabdUgaRbqabaGccqGGSaalcaWLjaGaaCzcamaabmaabaGaeGOmaiJaeG4mamdacaGLOaGaayzkaaaaleaacqaIWaamaeaacqaIXaqma0Gaey4kIipaaaa@4AB4@

where *δ*_*jk *_is the Kronecker delta function. The functional form of the polynomials are:

p1(x)=α13(2x−1),p2(x)=α25(6x2−6x+1),p3(x)=α37(2x−1)(10x2−10x+1),p4(x)=α49(210x4−420x3+270x2−60x+3),p5(x)=α511(252x5−630x4+560x3−210x2+30x−1,)p6(x)=α613(924x6−2772x5+3150x4−1680x3+420x2−42x+1),     (24)
 MathType@MTEF@5@5@+=feaafiart1ev1aaatCvAUfKttLearuWrP9MDH5MBPbIqV92AaeXatLxBI9gBaebbnrfifHhDYfgasaacH8akY=wiFfYdH8Gipec8Eeeu0xXdbba9frFj0=OqFfea0dXdd9vqai=hGuQ8kuc9pgc9s8qqaq=dirpe0xb9q8qiLsFr0=vr0=vr0dc8meaabaqaciaacaGaaeqabaqabeGadaaakeaafaqaaeGbbaaaaeaacqWGWbaCdaWgaaWcbaGaeGymaedabeaakmaabmaabaGaemiEaGhacaGLOaGaayzkaaGaeyypa0dcciGae8xSde2aaSbaaSqaaiabigdaXaqabaGcdaGcaaqaaiabiodaZaWcbeaakmaabmaabaGaeGOmaiJaemiEaGNaeyOeI0IaeGymaedacaGLOaGaayzkaaGaeiilaWcabaGaemiCaa3aaSbaaSqaaiabikdaYaqabaGcdaqadaqaaiabdIha4bGaayjkaiaawMcaaiabg2da9iab=f7aHnaaBaaaleaacqaIYaGmaeqaaOWaaOaaaeaacqaI1aqnaSqabaGcdaqadaqaaiabiAda2iabdIha4naaCaaaleqabaGaeGOmaidaaOGaeyOeI0IaeGOnayJaemiEaGNaey4kaSIaeGymaedacaGLOaGaayzkaaGaeiilaWcabaGaemiCaa3aaSbaaSqaaiabiodaZaqabaGcdaqadaqaaiabdIha4bGaayjkaiaawMcaaiabg2da9iab=f7aHnaaBaaaleaacqaIZaWmaeqaaOWaaOaaaeaacqaI3aWnaSqabaGcdaqadaqaaiabikdaYiabdIha4jabgkHiTiabigdaXaGaayjkaiaawMcaamaabmaabaGaeGymaeJaeGimaaJaemiEaG3aaWbaaSqabeaacqaIYaGmaaGccqGHsislcqaIXaqmcqaIWaamcqWG4baEcqGHRaWkcqaIXaqmaiaawIcacaGLPaaacqGGSaalaeaacqWGWbaCdaWgaaWcbaGaeGinaqdabeaakmaabmaabaGaemiEaGhacaGLOaGaayzkaaGaeyypa0Jae8xSde2aaSbaaSqaaiabisda0aqabaGcdaGcaaqaaiabiMda5aWcbeaakmaabmaabaGaeGOmaiJaeGymaeJaeGimaaJaemiEaG3aaWbaaSqabeaacqaI0aanaaGccqGHsislcqaI0aancqaIYaGmcqaIWaamcqWG4baEdaahaaWcbeqaaiabiodaZaaakiabgUcaRiabikdaYiabiEda3iabicdaWiabdIha4naaCaaaleqabaGaeGOmaidaaOGaeyOeI0IaeGOnayJaeGimaaJaemiEaGNaey4kaSIaeG4mamdacaGLOaGaayzkaaGaeiilaWcabaGaemiCaa3aaSbaaSqaaiabiwda1aqabaGcdaqadaqaaiabdIha4bGaayjkaiaawMcaaiabg2da9iab=f7aHnaaBaaaleaacqaI1aqnaeqaaOWaaOaaaeaacqaIXaqmcqaIXaqmaSqabaGcdaqadaqaaiabikdaYiabiwda1iabikdaYiabdIha4naaCaaaleqabaGaeGynaudaaOGaeyOeI0IaeGOnayJaeG4mamJaeGimaaJaemiEaG3aaWbaaSqabeaacqaI0aanaaGccqGHRaWkcqaI1aqncqaI2aGncqaIWaamcqWG4baEdaahaaWcbeqaaiabiodaZaaakiabgkHiTiabikdaYiabigdaXiabicdaWiabdIha4naaCaaaleqabaGaeGOmaidaaOGaey4kaSIaeG4mamJaeGimaaJaemiEaGNaeyOeI0IaeGymaeJaeiilaWcacaGLOaGaayzkaaaabaGaemiCaa3aaSbaaSqaaiabiAda2aqabaGcdaqadaqaaiabdIha4bGaayjkaiaawMcaaiabg2da9iab=f7aHnaaBaaaleaacqaI2aGnaeqaaOWaaOaaaeaacqaIXaqmcqaIZaWmaSqabaGcdaqadaqaaiabiMda5iabikdaYiabisda0iabdIha4naaCaaaleqabaGaeGOnaydaaOGaeyOeI0IaeGOmaiJaeG4naCJaeG4naCJaeGOmaiJaemiEaG3aaWbaaSqabeaacqaI1aqnaaGccqGHRaWkcqaIZaWmcqaIXaqmcqaI1aqncqaIWaamcqWG4baEdaahaaWcbeqaaiabisda0aaakiabgkHiTiabigdaXiabiAda2iabiIda4iabicdaWiabdIha4naaCaaaleqabaGaeG4mamdaaOGaey4kaSIaeGinaqJaeGOmaiJaeGimaaJaemiEaG3aaWbaaSqabeaacqaIYaGmaaGccqGHsislcqaI0aancqaIYaGmcqWG4baEcqGHRaWkcqaIXaqmaiaawIcacaGLPaaacqGGSaalaaGaaCzcaiaaxMaadaqadaqaaiabikdaYiabisda0aGaayjkaiaawMcaaaaa@FDAD@

where the *α*_*i*_'s are applied to each functional value and represent uniform random variables drawn from the interval [0.5,1.5]. The remaining 5,400 rows are populated with random numbers drawn from a uniform distribution on the interval [-3,3]. Figure 3 provides an illustration of the polynomials in the presence of Gaussian noise (*σ *= 0.25).

**Figure 4:**
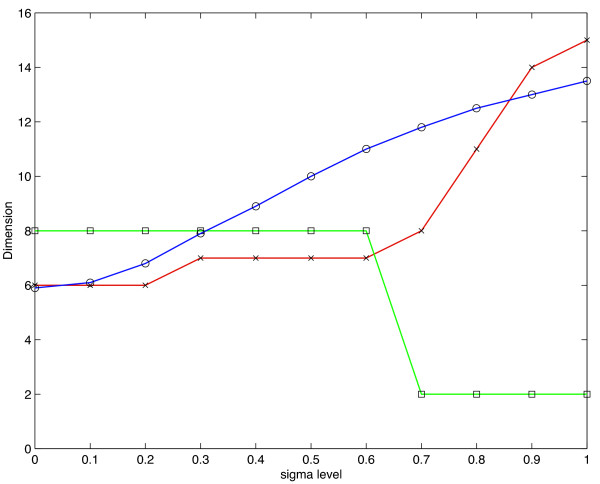
**Predicted dimension for simulated data**. Predicted dimension versus noise Level. Shown is the information dimension (line with circle markers), Jolliffe's modification of the Guttman-Kaiser rule (line with star markers) and LEV (line with square markers) plotted against noise level, measured in standard deviations. Note that the predictions given by both the information dimension and Guttman-Kaiser's rule increase as the noise level increases, while LEV drops sharply (see Text).

A singular value decomposition was performed on *X*_*σ*_, for *σ *ranging from 0.0 to 1.0 by 0.1 increments. In the absence of Gaussian noise (*σ *= 0), the information dimension predicts the dimension of the data to be *n*_0 _= 5.9, which compares favorably with the true dimension of 6. It should be noted, however, that like other stopping criteria, the information dimension is a function of the noise present in the data. Figure 4 illustrates this dependence when the number of assays is 15. The information dimension (line with circle markers), Jolliffe's modification of the Guttman-Kaiser rule (line with star markers) and LEV (line with square markers) are plotted against noise level, measured in standard deviations. The predictions given by both the information dimension and Guttman-Kaiser's rule increase as the noise level increases, while LEV drops sharply. The reason LEV decreases is that higher noise levels cause the distribution of the eigenvalues to look uniform. The results of applying all of the stopping techniques to the matrix *X*_*σ *_for *σ *= 0 and *σ *= 0.25 are summarized in Table 2.

**Table 2:** Summary of results

Column No.	1	2	3	4	5	6	7	8
Data Set	*Y *+ *N*_0_	*Y *+ *N*_.25_	alpha	cdc15	elutriation	fibro	sporula	tumor
Broken Stick, (BS)	1	1	2	4	3	2	1	3
Modified BS	1	1	2	4	3	2	1	3
Velicer's MAP	2/8	2	3	5	3	3	2	8/10
Kaiser-Guttman, (KG)	2	2	3	4	3	3	2	9
Jolliffe's KG	7	7	4	5	4	3	3	14
LEV Diagram	6/8	6/8	4/5	5	4/5	4/6	3	12/21
Parallel Analysis	1	1	5	4	3	3	2	8
Scree Test	8	8	5	5	4	6	4	7
Info Dimension	5.9	7.3	11.1	7.2	6.4	3.0	2.6	17.3

Gleason-Staelin Stat	.525	.45	.34	.37	.38	.54	.58	.37
Normalized Entropy	.917	.941	.779	.726	.706	.438	.493	.696

80% of Var.	4	4	9	4	5	3	3	19
90% of Var.	7	7	14	7	9	5	3	33
Bartlett's Test	15	15	22	15	14	12	7	60

The table contains the results of twelve stopping rules along with two measures of data information content for six cDNA microarray data sets. We recommend looking for a consensus among the rules given in the upper portion of the table, while avoiding rules based on cumulative percent of variation explained or Barlett's test. Synthetic data sets are summarized in columns 1 (no noise) and 2 (Gaussian noise, *μ *= 0 and *σ *= 0.25). The matrix sizes for columns 1 through 8 are : (6000 × 15), (6000 × 15), (4579 × 22), (799 × 15), (5981 × 14), (517 × 12), (6118 × 7), and (1375 × 60).

### 3.2 Yeast cdc15 cell-cycle data set

A PCA was performed on the genes identified in Spellman [34] responsible for cell cycle regulation in yeast samples. The cdc15 data set contains *p *= 799 rows (genes) and *q *= 15 columns representing equally spaced time points. The unpolished data set appears to have a high information content as suggested by the normalized entropy which is .7264 and the Gleason-Staelin statistic which is 0.3683. Therefore, we should expect the stopping criteria to indicate that significant dimension reduction may not be possible.

Eigenvalues based on both the covariance and correlation matrices are given in Table 2. From the given data we see that it requires the first seven eigenvalues to account for over 90% of the variance in the data. The Kaiser-Guttman test retains the first four eigenvalues which represents the number of eigenvalues obtained from the correlation matrix that exceeds unity. To incorporate the effect of sample variance Jolliffe [9] suggests that the appropriate number to retain are those eigenvalues whose value exceed 0.7. Jolliffe's modification of Kaiser-Guttman would indicate that the first five eigenvalues are significant. Parallel analysis compares the eigenvalues obtained from either the correlation or covariance matrix of the data to those obtain from a matrix whose entries are drawn from a uniform random distribution.

**Figure 5:**
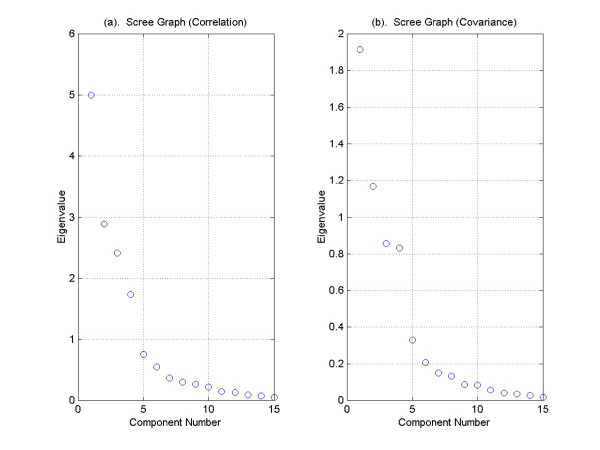
**Cattell's scree test**. Cattell's scree test using eigenvalues obtained from (a) the correlation matrix and from (b) the covariance matrix of the yeast cdc15 cell-cycle data set. Since the first inflection point occurs between the fourth and fifth eigenvalues, the implied dimension is five. Cattell's scree test using eigenvalues obtained from (a) the correlation matrix and from (b) the covariance matrix of the yeast cdc15 cell-cycle data set. Since the first inflection point occurs between the fourth and fifth eigenvalues, the implied dimension is five.

Cattell's scree test looks for an inflection point in the graph of the eigenvalues, which are plotted in descending order. Figure 5 illustrates Cattell's scree test for eigenvalues obtained from the correlation matrix and from the covariance matrix respectively. By inspecting the differences of the differences between eigenvalues, we see that the first inflection point occurs between the fourth and fifth eigenvalues. Therefore, the scree test gives a dimension of five.

**Figure 6:**
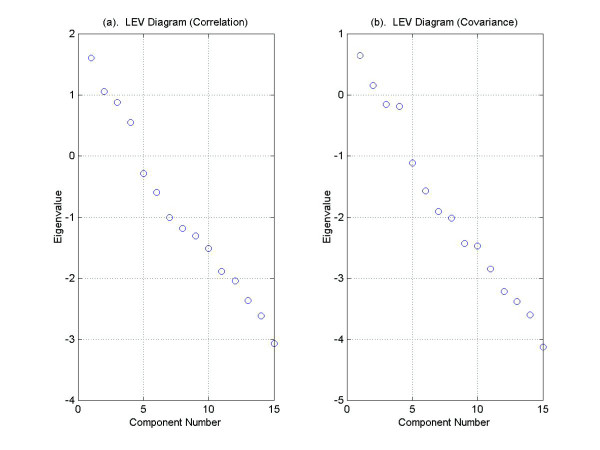
**The LEV Diagram**. LEV Diagram using eigenvalues obtained from (a) the correlation matrix and from (b) the covariance matrix of the yeast cdc15 cell-cycle data set. The fifth through fifteen eigenvalues lie approximately on a line indicating a dimension of five.

Figure 6 contains graphs of the Log-eigenvalue diagram, LEV, where the eigenvalues are obtained from the correlation matrix (Figure 6.a) and the covariance matrix (Figure 6.b). For each eigenvalue *λ*_*j*_, we graph log (*λ*_*j*_) against *j *and look for the point at which the eigenvalues decay linearly. The method is based on the conjecture that the eigenvalues associated with eigenvectors that are dominated by noise will decay geometrically. The LEV diagram is subject to interpretation and may suggest retaining 0, 3, or 9 components.

**Figure 7:**
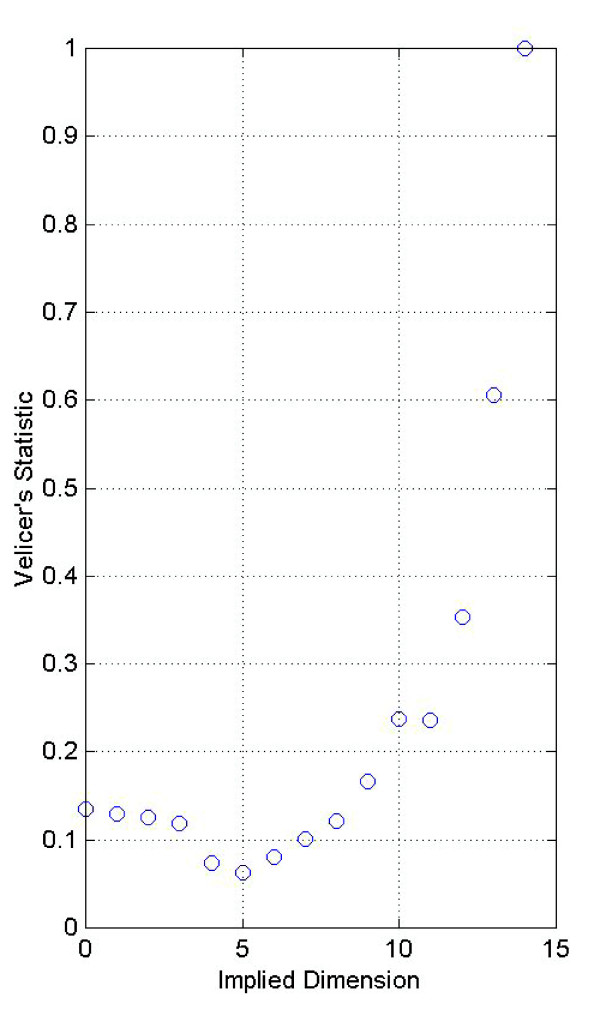
**Velicer's minimum average partial statistic**. Velicer's minimum average partial statistic displays a minimum value at five, indicating that the implied dimension is five.

Figure 7 illustrates Velicer's minimum average partial correlation statistic. It is based upon the average of the squared partial correlations between *q *variables after the first *m *components have been removed [52]. The summary statistic is given by

fm=1q(q−1)∑∑i≠j(rij∗)2,     (25)
 MathType@MTEF@5@5@+=feaafiart1ev1aaatCvAUfKttLearuWrP9MDH5MBPbIqV92AaeXatLxBI9gBaebbnrfifHhDYfgasaacH8akY=wiFfYdH8Gipec8Eeeu0xXdbba9frFj0=OqFfea0dXdd9vqai=hGuQ8kuc9pgc9s8qqaq=dirpe0xb9q8qiLsFr0=vr0=vr0dc8meaabaqaciaacaGaaeqabaqabeGadaaakeaacqWGMbGzdaWgaaWcbaGaemyBa0gabeaakiabg2da9maalaaabaGaeGymaedabaGaemyCae3aaeWaaeaacqWGXbqCcqGHsislcqaIXaqmaiaawIcacaGLPaaaaaWaaabqaeaadaaeqbqaamaabmaabaGaemOCai3aa0baaSqaaiabdMgaPjabdQgaQbqaaiabgEHiQaaaaOGaayjkaiaawMcaamaaCaaaleqabaGaeGOmaidaaOGaeiilaWIaaCzcaiaaxMaadaqadaqaaiabikdaYiabiwda1aGaayjkaiaawMcaaaWcbaGaemyAaKMaeyiyIKRaemOAaOgabeqdcqGHris5aaWcbeqab0GaeyyeIuoaaaa@4E26@

where rij∗
 MathType@MTEF@5@5@+=feaafiart1ev1aaatCvAUfKttLearuWrP9MDH5MBPbIqV92AaeXatLxBI9gBaebbnrfifHhDYfgasaacH8akY=wiFfYdH8Gipec8Eeeu0xXdbba9frFj0=OqFfea0dXdd9vqai=hGuQ8kuc9pgc9s8qqaq=dirpe0xb9q8qiLsFr0=vr0=vr0dc8meaabaqaciaacaGaaeqabaqabeGadaaakeaacqWGYbGCdaqhaaWcbaGaemyAaKMaemOAaOgabaGaey4fIOcaaaaa@31ED@ is the element in the *i*-th row and *j*-th column of the matrix of partial correlations and co-variances. The pattern of the statistic given in Figure 7 for cdc15 cell cycle data is typical in that the statistic first declines then rises. Once the statistic begins to rise it indicates that additional principal components represent more variance than covariance [7]. Therefore, no components are retained after the average squared partial correlation reaches a minimum. Here the minimum occurs at *j *= 5, which suggests retaining the first five principal components.

**Figure 8:**
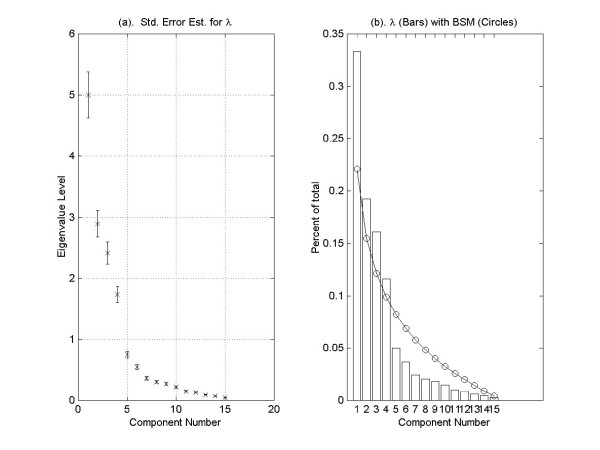
**The cdc15 yeast cell-cycle data set**. (a) Error bars about the eigenvalues obtained from the covariance matrix of the cdc15 yeast cell-cycle data set illustrating North *et al. *(1982) "rule of thumb" estimate with *δ *= 1.5. The spacing between the second and third eigenvalues indicate a possible degenerate subspace spanned by the associated eigenvectors, (b) is a graph of the broken stick model (circles) plotted against the eigenvalues (bars) obtained from the covariance matrix of the yeast cdc15 data set. The broken stick model (and the modified broken stick model) indicate a dimension of four.

Figure 8. a shows the eigenvalues from the covariance matrix along with error bars representing the sampling error estimate suggested by North *et al*. [38] and presented in Section 2.3 above. Figure 8.b shows the eigenvalues obtained from the covariance matrix superimposed on the broken stick distribution. Applying the rule of thumb method given in North *et al*. [38], we find that the first six eigenvalues are sufficiently separated and may be treated as individual subspaces. The remaining eigenvalues are close compared to their sampling error. Therefore, when applying the broken stick model we require that the total variance of the effectively degenerate subspace spanned by the associated eigenvectors to exceed the value suggested by the broken stick model for the sum of the seventh through fifteen lengths. This would also suggest that we accept or reject the entire subspace. Of course the tail of the distribution can never exceed that suggested by the broken stick distribution. The broken stick model suggests a dimension of four for the cdc15 data.

### 3.3 Summary of results For six microarray data sets

Table 2 summarizes the results of the stopping criteria for six microarray data sets. Note that Bartlett's test fails to discard any components. The null hypothesis that all roots are equal is rejected at every stage of the test. The large sample size of each data set was a major factor for all roots testing out to be significantly different. The broken stick model consistently retained the fewest number of components, which appears consistent with comments in the literature. The results of the modified broken stick model were identical to that of the original model since the first few eigenvalues in each data set appear to be well separated, at least with respect to Equation (6). Since no effective degenerate subspaces were identified, all subspaces matched those of the original model. The cumulative percent of variance at the 90% level retains the greatest number of components while components retained at the 80% level appear to be more consistent with other rules. Regardless of the cutoff level chosen, this method is completely arbitrary and appears to be without merit. While the LEV diagram is less subjective, it is often difficult to interpret. Kaiser-Guttman, Jolliffe's modification of Kaiser-Guttman, Cattell's scree test, parallel analysis and Velicer's MAP consistently retained similar numbers of components. The information dimension gave comparable results. It often retained the most components of the five aforementioned rules suggesting that it may provides an upper bound on the interpretable number of components.

## 4 Conclusion

Principal component analysis is a powerful descriptive and data reduction technique for the analysis of microarray data. Twelve stopping rules to determine the appropriate level of data reduction were presented including a new heuristic model based on statistical entropy. While the issue of component retention remains unresolved, the information dimension provides a reasonable and conservative estimate of the upper bound of the true dimension of the data.

Our analysis shows that the broken stick model and Velicer's MAP consistently retained the fewest number of components while stopping rules based on percent variation explained and Bartlett's test retained the greatest number of components. We do not recommend the use of Bartlett's test (as presented here) or those based on the cumulative percent of variation explained. Due to the large sample size (typical of microarray data), Bartlett's test failed to discard any components, while rules based on percent variation explained are completely arbitrary in nature.

For the analysis of cDNA microarray data, we do not recommend any one stopping technique. Instead, we suggest that one look for a "consensus dimension" given by the modified broken stick model, Velicer's MAP, Jolliffe modification of Kaiser-Guttman, the LEV diagram, parallel analysis, the scree test and the information dimension while using the information dimension as an upper bound for the number of components to retain. Computing all seven stopping rules is an easy task and Matlab routines are available from the authors.

As a guiding example consider the results from the cdc15 cDNA data set given in Table 3. For the cdc15 data set, the consensus is split between four and five; however, given that the information dimension is seven, it appears reasonable to choose five as the appropriate dimension in which to work.

**Table 3:** Eigenvalues of yeast cdc15 cell cycle data

No.	Eigenvalue (Covariance)	Percent of Total	Cumulated Percentage	Eigenvalue (Correlation)	Random
1	1.9162	32.25	32.25	5.0025	0.1004
2	1.1681	19.66	51.91	2.8896	0.0997
3	0.8560	14.41	66.32	2.4140	0.0975
4	0.8320	14.00	80.32	1.7371	0.0940
5	0.3295	5.55	85.87	0.7499	0.0905
6	0.2087	3.51	89.38	0.5495	0.0870
7	0.1490	2.51	91.89	0.3663	0.0841
8	0.1337	2.25	94.14	0.3064	0.0831
9	0.0881	1.48	95.62	0.2706	0.0808
10	0.0842	1.42	97.04	0.2206	0.0801
11	0.0580	0.98	98.02	0.1507	0.0779
12	0.0402	0.68	98.69	0.1292	0.0750
13	0.0341	0.57	99.27	0.0930	0.0727
14	0.0273	0.46	99.73	0.0731	0.0702
15	0.0162	0.27	100.00	0.0464	0.0650

## A A brief overview of stopping techniques

### A.1 Introduction

Franklin *et al. *[23] suggests that when a researcher uses PCA for data analysis, the most critical problem faced is determining the number of components to retain. Indeed, retaining too many components potentially leads to an attempt to ascribe physical meaning to what may be nothing more than noise in the data set, while retaining too few components may cause the researcher to discard valuable information. Many methods have been proposed to address the question of component selection and a brief review is given here. A more extensive review can be found in [[7], Section 2.8]; [[9], Section 6.1]; [[6], Chapter 5]. These methods may be categorized as either heuristic or statistical approaches [20]. The statistical methods may be further partitioned into two groups: those that make assumptions regarding the distribution of the data and those that do not make such assumptions. Jolliffe [9] criticizes the former stating that the distributional assumptions are often unrealistic and adds that these methods tend to over-estimate the number of components. The latter methods tend to be computationally intensive (for example, cross validation and bootstrapping). Our approach is to consider only heuristic methods here with the exception of Velicer's Partial Correlation test, which is a statistical method that does not require distributional assumptions nor is it computationally intensive. We present below a brief discussion of the heuristic techniques for determining the dimensionality of data sets as well as Velicer's Partial Correlation test.

### A. 2 Scree test

The scree test is a graphical technique attributed to Cattell [49] who described it in term of retaining the correct number of factors in a factor analysis. However, it is widely used in PCA [9]. While a scree graph is simple to construct, its interpretation may be highly subjective. Let *λ*_*k *_represent the *k*-th eigenvalue obtained from a covariance or correlation matrix. A graph of *λ*_*k *_against *k *is known as a scree graph. The location on the graph where a sharp change in slope occurs in the line segments joining the points is referred to as an elbow. The value of *k *at which this occurs represents the number of components that should be retained in the PCA. Jackson [7] notes that the scree test is a graphical substitute for a significance test. He points out that interpretation might be confounded in cases where the scree graph either does not have a clearly defined break or has more than one break. Also, if the first few roots are widely separated, it may be difficult to interpret where the elbow occurred due to a loss in detail caused by scaling. This problem might be remedied using the LEV described below.

### A.3 Proportion of total variance explained

In a PCA model, each eigenvalue represents the level of variation explained by the associated principal component. A simple and popular stopping rule is based on the proportion of the total variance explained by the principal components retained in the model. If *k *components are retained, then we may represent the cumulative variance explained by the first *k *PC's by

tk=∑λitrace(S)     (26)
 MathType@MTEF@5@5@+=feaafiart1ev1aaatCvAUfKttLearuWrP9MDH5MBPbIqV92AaeXatLxBI9gBaebbnrfifHhDYfgasaacH8akY=wiFfYdH8Gipec8Eeeu0xXdbba9frFj0=OqFfea0dXdd9vqai=hGuQ8kuc9pgc9s8qqaq=dirpe0xb9q8qiLsFr0=vr0=vr0dc8meaabaqaciaacaGaaeqabaqabeGadaaakeaacqWG0baDdaWgaaWcbaGaem4AaSgabeaakiabg2da9maalaaabaWaaabqaeaaiiGacqWF7oaBdaWgaaWcbaGaemyAaKgabeaaaeqabeqdcqGHris5aaGcbaGaemiDaqNaemOCaiNaemyyaeMaem4yamMaemyzau2aaeWaaeaacqWGtbWuaiaawIcacaGLPaaaaaGaaCzcaiaaxMaadaqadaqaaiabikdaYiabiAda2aGaayjkaiaawMcaaaaa@4459@

where *S *is the sample covariance matrix. The researcher decides on a satisfactory value for *t*(*k*) and then determines *k *accordingly. The obvious problem with the technique is deciding on an appropriate *t*(*k*). In practice it is common to select levels between 70% to 95% [9]. Jackson [7] argues strongly against the use of this method except possibly for exploratory purposes when little is known about the population of the data. An obvious problem occurs when several eigenvalues are of similar magnitude. For example, suppose for some *k *= k_*_, *t*(k_*_) = 0.50 and the remaining *q *- *k *eigenvalues have approximately the same magnitude. Can one justify adding more components until some predetermined value of *t*(*k*) is reached? Jolliffe [9] points out that the rule is equivalent to looking at the spectral decomposition of *S*. Determining how many terms to include in the decomposition is closely related to *t*(*k*) because an appropriate measure of a lack-of-fit is ∑i=k+1qλi
 MathType@MTEF@5@5@+=feaafiart1ev1aaatCvAUfKttLearuWrP9MDH5MBPbIqV92AaeXatLxBI9gBaebbnrfifHhDYfgasaacH8akY=wiFfYdH8Gipec8Eeeu0xXdbba9frFj0=OqFfea0dXdd9vqai=hGuQ8kuc9pgc9s8qqaq=dirpe0xb9q8qiLsFr0=vr0=vr0dc8meaabaqaciaacaGaaeqabaqabeGadaaakeaadaaeWaqaaGGaciab=T7aSnaaBaaaleaacqWGPbqAaeqaaaqaaiabdMgaPjabg2da9iabdUgaRjabgUcaRiabigdaXaqaaiabdghaXbqdcqGHris5aaaa@38E2@ (see Jolliffe 2002, pp. 113).

### A.4 Average eigenvalue (Guttman-Kaiser rule and Jolliffe's Rule)

The most common stopping criterion in PCA is the Guttman-Kaiser criterion [7]. Principal components associated with eigenvalues derived from a covariance matrix, and that are larger in magnitude than the average of the eigenvalues, are retained. In the case of eigenvalues derived from a correlation matrix, the average is one. Therefore, any principal component associated with an eigenvalue whose magnitude is greater than one is retained.

Based on simulation studies, Jolliffe [9] modified this rule using a cut-off of 70% of the average root to allow for sampling variation. Rencher [27] states that this method works well in practice but when it errs, it is likely to retain too many components. It is also noted that in cases where the data set contains a large number of variables that are not highly correlated, the technique tends to over estimate the number of components. Table 4 lists eigenvalues in descending order of magnitude from the correlation matrix associated with a (300 × 9) random data matrix. The elements of the random matrix were drawn uniformly over the interval [0, 1] and a PCA performed on the correlation matrix. Note that the first four eigenvalues have values that exceed 1 and all nine eigenvalues have values that exceed 0.7. Thus, Kaiser's rule and its modification suggest the existence of "significant PCs" from randomly generated data – a criticism that calls into question its validity [20,25,50,51].

**Table 4:** Eigenvalues from a random matrix.

No.	1	2	3	4	5	6	7	8	9
Eigenvalue	1.21	1.20	1.13	1.03	0.96	0.93	0.89	0.86	0.77

### A.5 Log-eigenvalue diagram, LEV

An adaptation of the scree graph is the log-eigenvalue diagram, where log(*λ*_*k*_) is plotted against *k*. It is based on the conjecture that eigenvalues corresponding to 'noise' should decay geometrically, therefore, those eigenvalues should appear linear. Farmer [50] investigated the procedure by studying LEV diagrams from different groupings of 6000 random numbers. He contends that the LEV diagram is useful in determining the dimension of the data.

### A.6 Velicer's partial correlation test

Velicer [52] proposed a test based on the partial correlations among the *q *original variables with one or more principal components removed. The criterion proposed is

fm=1q(q−1)∑∑i≠j(rij*)2,     (27)
 MathType@MTEF@5@5@+=feaafiart1ev1aaatCvAUfKttLearuWrP9MDH5MBPbIqV92AaeXatLxBI9gBaebbnrfifHhDYfgasaacH8akY=wiFfYdH8Gipec8Eeeu0xXdbba9frFj0=OqFfea0dXdd9vqai=hGuQ8kuc9pgc9s8qqaq=dirpe0xb9q8qiLsFr0=vr0=vr0dc8meaabaqaciaacaGaaeqabaqabeGadaaakeaacqWGMbGzdaWgaaWcbaGaemyBa0gabeaakiabg2da9maalaaabaGaeGymaedabaGaemyCae3aaeWaaeaacqWGXbqCcqGHsislcqaIXaqmaiaawIcacaGLPaaaaaWaaabqaeaadaaeqbqaamaabmaabaGaemOCai3aa0baaSqaaiabdMgaPjabdQgaQbqaaiabcQcaQaaaaOGaayjkaiaawMcaamaaCaaaleqabaGaeGOmaidaaOGaeiilaWIaaCzcaiaaxMaadaqadaqaaiabikdaYiabiEda3aGaayjkaiaawMcaaaWcbaGaemyAaKMaeyiyIKRaemOAaOgabeqdcqGHris5aaWcbeqab0GaeyyeIuoaaaa@4E17@

where rij∗
 MathType@MTEF@5@5@+=feaafiart1ev1aaatCvAUfKttLearuWrP9MDH5MBPbIqV92AaeXatLxBI9gBaebbnrfifHhDYfgasaacH8akY=wiFfYdH8Gipec8Eeeu0xXdbba9frFj0=OqFfea0dXdd9vqai=hGuQ8kuc9pgc9s8qqaq=dirpe0xb9q8qiLsFr0=vr0=vr0dc8meaabaqaciaacaGaaeqabaqabeGadaaakeaacqWGYbGCdaqhaaWcbaGaemyAaKMaemOAaOgabaGaey4fIOcaaaaa@31ED@ is the partial correlation between the *i*-th and *j*-th variables. Jackson [7] notes that the logic behind Velicer's test is that as long as *f*_*k *_is decreasing, the partial correlations are declining faster than the residual variances. This means that the test will terminate when, on the average, additional principal components would represent more variance than covariance. Jolliffe [9] warns that the procedure is plausible for use in a factor analysis, but may underestimate the number of principal components in a PCA. This is because it will not retain principal components dominated by a single variable whose correlations with other variables are close to zero.

### A.7 Bartlett's equality of roots test

It has been argued in the literature (see North, [38]) that eigenvalues that are equal to each other should be treated as a unit, that is, they should either all be retained or all discarded. A stopping rule can be formulated where the last m eigenvalues are tested for equality. Jackson [7] presents a form of a test developed by Bartlett [53] which is

χ2=−ν∑j−k+1qln⁡(λj)+ν(q−k)ln⁡[∑j=k+1qλjq−k]     (28)
 MathType@MTEF@5@5@+=feaafiart1ev1aaatCvAUfKttLearuWrP9MDH5MBPbIqV92AaeXatLxBI9gBaebbnrfifHhDYfgasaacH8akY=wiFfYdH8Gipec8Eeeu0xXdbba9frFj0=OqFfea0dXdd9vqai=hGuQ8kuc9pgc9s8qqaq=dirpe0xb9q8qiLsFr0=vr0=vr0dc8meaabaqaciaacaGaaeqabaqabeGadaaakeaaiiGacqWFhpWydaahaaWcbeqaaiabikdaYaaakiabg2da9iabgkHiTiab=17aUnaaqahabaGagiiBaWMaeiOBa42aaeWaaeaacqWF7oaBdaWgaaWcbaGaemOAaOgabeaaaOGaayjkaiaawMcaaiabgUcaRiab=17aUnaabmaabaGaemyCaeNaeyOeI0Iaem4AaSgacaGLOaGaayzkaaGagiiBaWMaeiOBa42aamWaaeaadaWcaaqaamaaqadabaGae83UdW2aaSbaaSqaaiabdQgaQbqabaaabaGaemOAaOMaeyypa0Jaem4AaSMaey4kaSIaeGymaedabaGaemyCaehaniabggHiLdaakeaacqWGXbqCcqGHsislcqWGRbWAaaaacaGLBbGaayzxaaaaleaacqWGQbGAcqGHsislcqWGRbWAcqGHRaWkcqaIXaqmaeaacqWGXbqCa0GaeyyeIuoakiaaxMaacaWLjaWaaeWaaeaacqaIYaGmcqaI4aaoaiaawIcacaGLPaaaaaa@6545@

where *χ*^2 ^has (1/2) (*q *- *k *- 1)(*q *- *k *- 2) degrees of freedom and *v *represents the number of degrees of freedom associated with the covariance matrix.

## Authors' contributions

R.C. and A.G. performed research and wrote the paper

## Reviewers' comments

### Orly Alter review

R. Cangelosi and A. Goriely present two novel mathematical methods for estimating the statistically significant dimension of a matrix. One method is based on the Shannon entropy of the matrix, and is derived from fundamental principles of information theory. The other method is a modification of the "broken stick" model, and is derived from fundamental principles of probability. Also presented are computational estimations of the dimensions of six well-studied DNA microarray datasets using these two novel methods as well as ten previous methods.

Estimating the statistically significant dimension of a given matrix is a key step in the mathematical modeling of data, e.g., as the authors note, for data interpretation as well as for estimating missing data. The question of how best to estimate the dimension of a matrix is still an open question. This open question is faced in most analyses of DNA microarray data (and other large-scale modern datasets). The work presented here is not only an extensive analysis of this open question. It is also the first work, to the best of my knowledge, to address this key open question in the context of DNA microarray data analysis. I expect it will have a significant impact on this field of research, and recommend its publication.

For example, R. Cangelosi and A. Goriely show that, in estimating the number of eigenvectors which are of statistical significance in the PCA analysis of DNA microarray data, the method of cumulative percent of variance should not be used. Unfortunately, this very method is used in an algorithm which estimates missing DNA microarray data by fitting the available data with cumulative-percent-of-variance- selected eigenvectors [Troyanskaya et al., Bioinformatics 17, 520 (2001)]. This might be one explanation for the superior performance of other PCA and SVD-based algorithms for estimating DNA microarray data [e.g., Kim et al., Bioinformatics 15, 187 (2005)].

In another example, R. Cangelosi and A. Goriely estimate that there are two eigenvectors which are of statistical significance in the yeast cdc15 cell-cycle dataset of 799 genes × 15 time points. Their mathematical estimation is in agreement with the previous biological experimental [Spellman et al., MBC 9, 3273 (1998)] as well as computational [Holter et al., PNAS 97, 8409 (2000)] interpretations of this dataset.

Declaration of competing interests: I declare that I have no competing interests.

### John Spouge's review (John Spouge was nominated by Eugene Koonin)

This paper reviews several methods based on principal component analysis (PCA) for determining the "true" dimensionality of a matrix subject to statistical noise, with specific application to microarray data. It also offers two new candidates for estimating the dimensionality, called "information dimension" and the " modified broken stick model".

Section 2.1 nicely summarizes matrix methods for reducing dimensionality in microarray data. It describes why PCA is preferable to a singular value decomposition (a change in the intensities of microarray data affects the singular value decomposition, but not PCA).

Section 2.2 analyzes the broken stick model. Section 2.3 explains in intuitive terms the authors' "modified broken stick model", but the algorithm became clear to me only when it was applied to data later in the paper. The broken stick model has the counterintuitive property of determining dimensionality without regard to the amount of data, implicitly ignoring the ability of increased data to improve signal-to-noise. The modified broken stick model therefore has some intuitive appeal.

Section 2.4 explains the authors' information dimension. The derivation is thorough, but the resulting measure is purely heuristic, as the authors point out. In the end, despite the theoretical gloss, it is a just formula, without any desirable theoretical properties or intuitive interpretation.

The evaluation of the novel measures therefore depends on their empirical performance, found in the Results and Discussion. Systematic responses to variables irrelevant to the (known) dimensionality of synthetic data become of central interest. In particular, the authors show data that their information dimension increases systematically with noise, clearly an undesirable property. The authors also test the dimensionality estimators on real microarray data. They conclude that six dimensionality measures are in rough accord, with three outliers: Bartlett's test, cumulative percent of variation explained, and the information dimension (which tends to be higher than other estimators). They therefore propose the information dimension as an upper bound for the true dimensionality, with a consensus estimate being derived from the remaining measures.

The choice of dimensionality measure is purely empirical. While it is desirable to check all estimators (and report them in general accord, if that is the case), it is undesirable to report all estimators for any large set of results. The information dimension's property of increasing with noise makes it undesirable as an estimator, and it can not be recommended. The main value of the paper therefore resides in its useful review and its software tools.

### Answers to John Spouge's review

The main point of the reviewer is the suggestion that the information dimension's undesirable property of increasing with noise makes it undesirable as an estimator. We analyze the information in detail and indeed reached the conclusion that its prediction increases with noise. In the preprint reviewed by Dr. Spouge, we only considered the effect of noise on the information dimension. It is crucial to note that ALL methods are functions of the noise level present in the data. In the new and final version of the manuscript, we study the effect of noise on two other methods (Jolliffe's modification of he Guttman-Kaiser rule and LEV). It clearly appears that in one case the estimator increases with noise and in the other one, it decreases with noise (both effects are undesirable and unavoidable). The message to the practitioner is the same, understand the signal to noise ratio of the data and act accordingly. We conclude that the information dimension could still be of interest as an estimator.

### David Horn and Roy Varshavsky joint review (both reviewers were nominated by O. Alter)

This paper discusses an important problem in data analysis using PCA. The term 'component retention' that the authors use in the title is usually referred to as dimensional truncation or, in more general terms, as data compression. The problem is to find the desired truncation level to assure optimal results for applications such as clustering, classification or various prediction tasks.

The paper contains a very exhaustive review of the history of PCA and describes many recipes for truncation proposed over the 100 years since PCA was introduced. The authors propose also one method of their own, based on the use of the entropy of correlation eigenvalues. A comparison of all methods is presented in Table 2, including 14 criteria applied to 6 microarray experiments. This table demonstrates that the results of their proposed 'information dimension' are very different from those of most other truncation methods.

We appreciate the quality of the review presented in this paper, and we recommend that it should be viewed and presented as such. But we have quite a few reservations regarding the presentation in general and their novel method in particular.

1. The motivation for dimensional reduction is briefly mentioned in the introduction, but this point is not elaborated later on in the paper. As a result, the paper lacks a target function according to which one could measure the performance of the various methods displayed in Table 2. We believe one should test methods according to how well they perform, rather than according to consensus. Performance can be measured on data, but only if a performance function is defined, e.g. the best Jaccard score achieved for classification of the data within an SVM approach. Clearly many other criteria can be suggested, and results may vary from one dataset to another, but this is the only valid scientific approach to decide on what methods should be used. We believe that it is necessary for the authors to discuss this issue before the paper is accepted for publication.

2. All truncation methods are heuristic. Also the new statistical method proposed here is heuristic, as the authors admit. An example presented in Table 1 looks nice, and should be regarded as some justification; however the novel method's disagreement with most other methods (in Table 2) raises the suspicion that the performance of the new method, once scrutinized by some performance criterion on real data, may be bad. The authors are aware of this point and they suggest using their method as an upper bound criterion, with which to decide if their proposed 'consensus dimension' makes sense. This, by itself, has a very limited advantage.

3. The abstract does not represent faithfully the paper. The new method is based on an 'entropy' measure but this is not really Shannon entropy because no probability is involved. It gives the impression that the new method is based on some 'truth' whereas others are ad-hoc which, in our opinion, is wrong (see item 2 above). We suggest that once this paper is recognized as a review paper the abstract will reflect the broad review work done here.

4. Some methods are described in the body of the article (e.g., broken stick model), while others are moved to the appendix (e.g., portion of total variance). This separation is not clear. Unifying these two sections can contribute to the paper readability.

In conclusion, since the authors admit that information dimension cannot serve as a stopping criterion for PCA compression, this paper should not be regarded as promoting a useful truncation method. Nevertheless, we believe that it may be very useful and informative in reviewing and describing the existing methods, once the modifications mentioned above are made. We believe this could then serve well the interested mathematical biology community.

### Answers to Horn's and Varshavsky's review

We would like to thank the reviewers for their careful reading of our manuscript and their positive criticisms. We have modified our manuscript and follow most of their recommendations.

Specifically, we answer each comment by the reviewer:

1. The status of the paper as a review or a regular article.

It is true that the paper contains a comprehensive survey of the literature and many references. Nevertheless, we believe that the article contains sufficiently many new results to be seen as a regular journal article. Both the modified broken-stick method and the information dimension are new and important results for the field of cDNA analysis.

2. The main criticism of the paper is that we did not test the performance of the different methods against some benchmark.

To answer this problem we performed extensive benchmarking of the different methods against noisy simulated data for which the true signal and its dimension was known. We have added this analysis in the paper where we provide an explicit example. This example clearly establishes our previous claim that the information dimension provides a useful upper bound for the true signal dimension (whereas other traditional methods such as Velicer's underestimate the true dimension). Upper bounds are extremely important in data analysis as they provide a reference point with respect to which other methods can be compared.

3. The abstract does not represent the paper.

We did modify the abstract to clarify the relationship of information dimension that we propose with respect to other methods (it is also a heuristic approach!). Now, with the added analysis and wording, we believe that the abstract is indeed a faithful representation of the paper.

4. Some methods appear in the appendix.

Indeed, the methods presented in the appendix are the one that we review. Since we present a modification of the broken-stick method along with a new heuristic technique, we believe it is appropriate to describe the broken-stick method in the main body of the text while relegating other known approaches (only used for comparison) to the appendix. Keeping in mind that this is a regular article rather than a review, we believe it is justified.
